# Supplementary material for: Targeting endogenous kidney regeneration using anti-IL11 therapy in acute and chronic models of kidney disease
Source: Nat Commun. 2022 Dec 5;13:7497. doi: 10.1038/s41467-022-35306-1 (PMC9723120; doi:10.1038/s41467-022-35306-1)

Supplementary Materials for

Targeting endogenous kidney regeneration using anti-IL11 therapy in acute and chronic models of kidney disease

Authors: Anissa A. Widjaja[1*], Sivakumar Viswanathan[1], Shamini G. Shekeran[1], Eleonora Adami[1,2], Wei-Wen Lim[1,3], Sonia Chothani[1], Jessie Tan[3], Joyce Goh Wei Ting[1], Chen Hui Mei[1], Sze Yun Lim[1], Carine M Boustany-Kari[4], Julie Hawkins[4], Enrico Petretto[1], Norbert Hubner[2,5,6], Sebastian Schafer[1], Thomas M. Coffman[1], Stuart A. Cook[1,3,7*]

*Corresponding author. Email: anissa.widjaja@duke-nus.edu.sg and stuart.cook@duke-nus.edu.sg

This file includes:

Supplementary Figures (1-12)

Supplementary Table 1

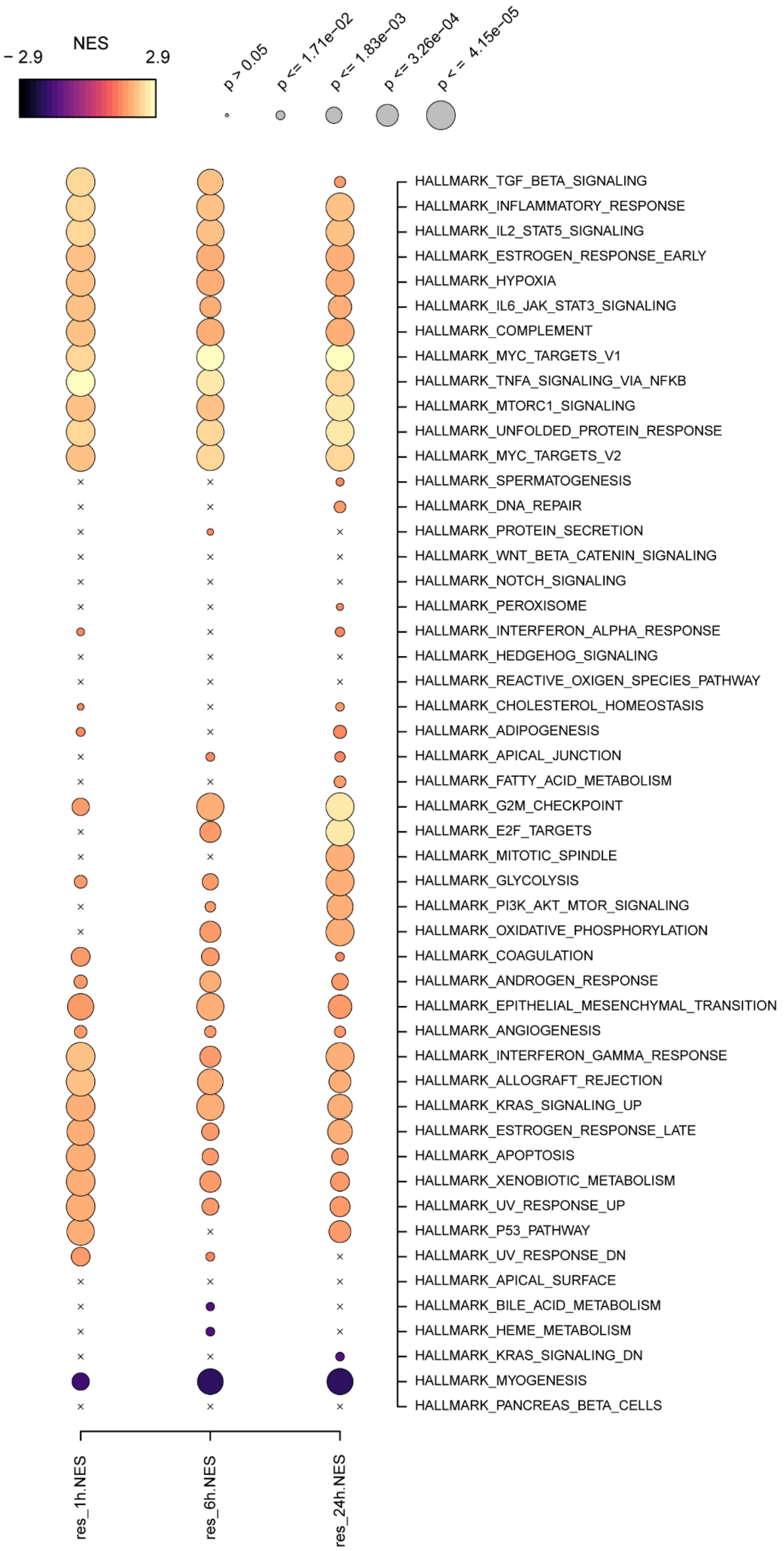

NES
− 2.9
2.9
p > 0.05
p <= 1.71e−02
p <= 1.83e−03
p <= 3.26e−04
p < = 4.15e−05
HALLMARK_TGF_BETA_SIGNALING
HALLMARK_INFLAMMATORY_RESPONSE
HALLMARK_IL2_STAT5_SIGNALING
HALLMARK_ESTROGEN_RESPONSE_EARLY
HALLMARK_HYPOXIA
HALLMARK_IL6_JAK_STAT3_SIGNALING
HALLMARK_COMPLEMENT
HALLMARK_MYC_TARGETS_V1
HALLMARK_TNFA_SIGNALING_VIA_NFKB
HALLMARK_MTORC1_SIGNALING
HALLMARK_UNFOLDED_PROTEIN_RESPONSE
HALLMARK_MYC_TARGETS_V2
HALLMARK_SPERMATOGENESIS
HALLMARK_DNA_REPAIR
HALLMARK_PROTEIN_SECRETION
HALLMARK_WNT_BETA_CATENIN_SIGNALING
HALLMARK_NOTCH_SIGNALING
HALLMARK_PEROXISOME
HALLMARK_INTERFERON_ALPHA_RESPONSE
HALLMARK_HEDGEHOG_SIGNALING
HALLMARK_REACTIVE_OXIGEN_SPECIES_PATHWAY
HALLMARK_CHOLESTEROL_HOMEOSTASIS
HALLMARK_ADIPOGENESIS
HALLMARK_APICAL_JUNCTION
HALLMARK_FATTY_ACID_METABOLISM
HALLMARK_G2M_CHECKPOINT
HALLMARK_E2F_TARGETS
HALLMARK_MITOTIC_SPINDLE
HALLMARK_GLYCOLYSIS
HALLMARK_PI3K_AKT_MTOR_SIGNALING
HALLMARK_OXIDATIVE_PHOSPHORYLATION
HALLMARK_COAGULATION
HALLMARK_ANDROGEN_RESPONSE
HALLMARK_EPITHELIAL_MESENCHYMAL_TRANSITION
HALLMARK_ANGIOGENESIS
HALLMARK_INTERFERON_GAMMA_RESPONSE
HALLMARK_ALLOGRAFT_REJECTION
HALLMARK_KRAS_SIGNALING_UP
HALLMARK_ESTROGEN_RESPONSE_LATE
HALLMARK_APOPTOSIS
HALLMARK_XENOBIOTIC_METABOLISM
HALLMARK_UV_RESPONSE_UP
HALLMARK_P53_PATHWAY
HALLMARK_UV_RESPONSE_DN
HALLMARK_APICAL_SURFACE
HALLMARK_BILE_ACID_METABOLISM
HALLMARK_HEME_METABOLISM
HALLMARK_KRAS_SIGNALING_DN
HALLMARK_MYOGENESIS
HALLMARK_PANCREAS_BETA_CELLS
res_1h.NES
res_6h.NES
res_24h.NES

Supplementary Figure 1: IL11 induces pro-inflammatory and epithelial-to-mesenchymal transcriptional responses in human tubular epithelial cells. Bubblemap showing results of hallmark gene set enrichment analysis for differentially expressed genes following IL11 stimulation over a time course (0, 1, 6, and 24 hours). Normalized enrichment scores for the hallmark gene sets were quantified using the fgsea R package with the fgsea-multilevel method that is based on an adaptive multilevel splitting Monte-Carlo approach. The quantified significance level was corrected using the Benjamini & Hochberg method. Normalized enrichment scores (NES) are represented by colors, from black (negative NES, suggesting down-regulation of the gene set) to yellow (positive NES, suggesting up-regulation). Dot size indicates significance, the larger the dot, the lower the adjusted p-value. Gene sets for the enrichment test were selected from the "H - Hallmark" collection in MSigDB. Source data are provided as a Source Data file.

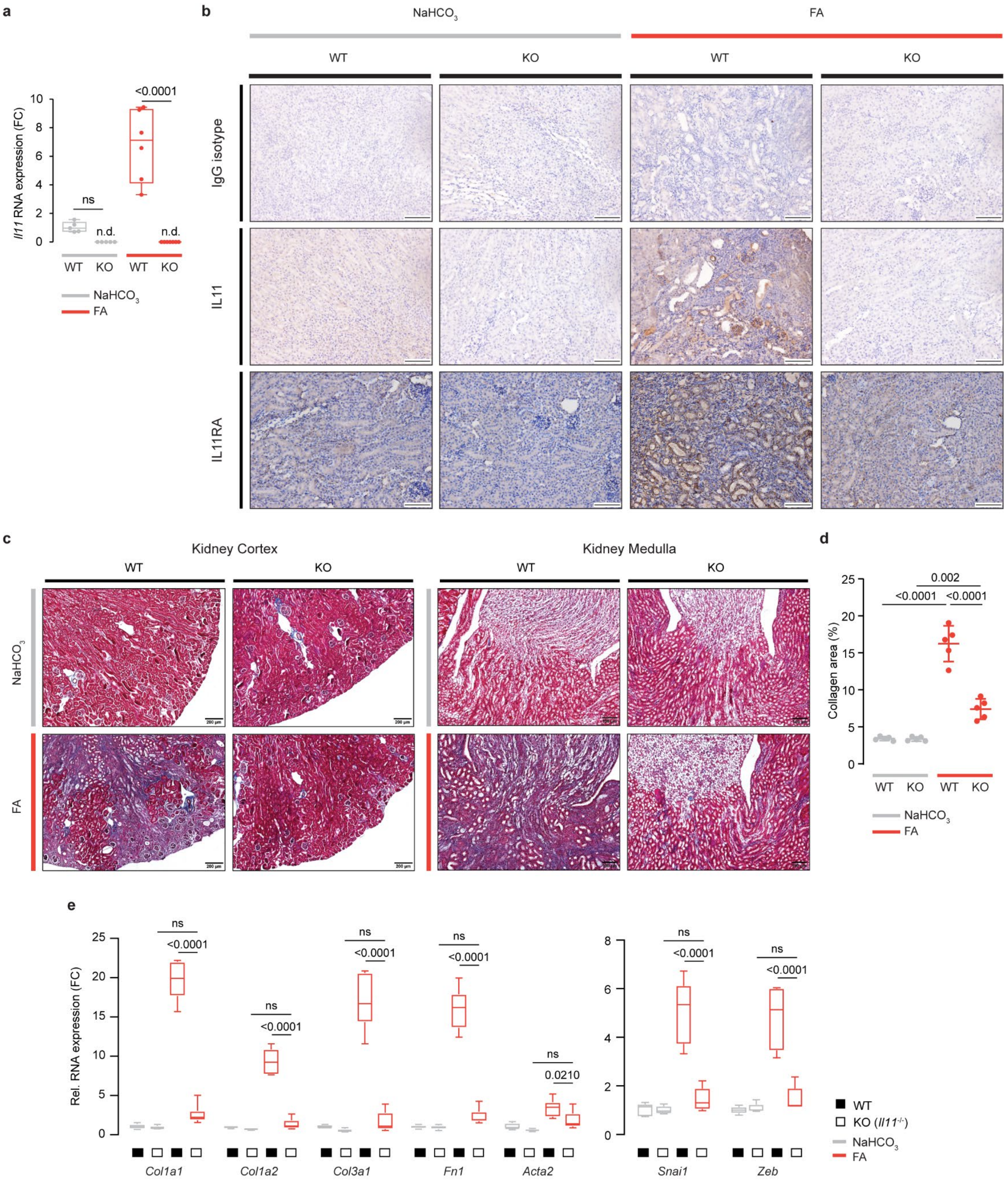

Supplementary Figure 2: *Il11* knockout mice are protected from renal inflammation and fibrosis. a Renal *Il11* RNA expression, **b** representative immunohistochemistry staining images of kidney stained for IL11 (with X203), IL11RA (with X209); staining with an IgG isotype control (11E10) is provided as negative control (representative dataset from n=3/group; scale bars: 100 μm), **c** representative Masson's Trichrome images of kidney cortex (scale bars: 200 μm) and medulla (scale bars: 100 μm) (representative dataset from n=5/group), **d** quantification of collagen area from Masson's Trichrome-stained kidney sections (n=5/group), **e** relative renal mRNA expression of fibrotic markers (*Col1a1, Col1a2, Col3a1, Fn1,*

Acta2), pro-inflammatory markers (*Tnfα*, *Il6, Ccl2*, *Ccl5, Il1β*), and pEMT markers (*Snai1*, and *Zeb*) from control and *Il11* KO mice post FA (Schematic Fig. 2a). **a, e** Data are shown as box-and-whisker with median (middle line), 25th–75th percentiles (box), and minimum-maximum values (whiskers); WT-$NaHC0_3$, *Il11*$^{-/-}$-$NaHC0_3$ (n=5/group), WT-FA (n=6), *Il11*$^{-/-}$-FA (n=7), **c** data are shown as mean±SD. **a, d, e** 2-way ANOVA with Sidak's correction. FC: Fold change. Source data are provided as a Source Data file.

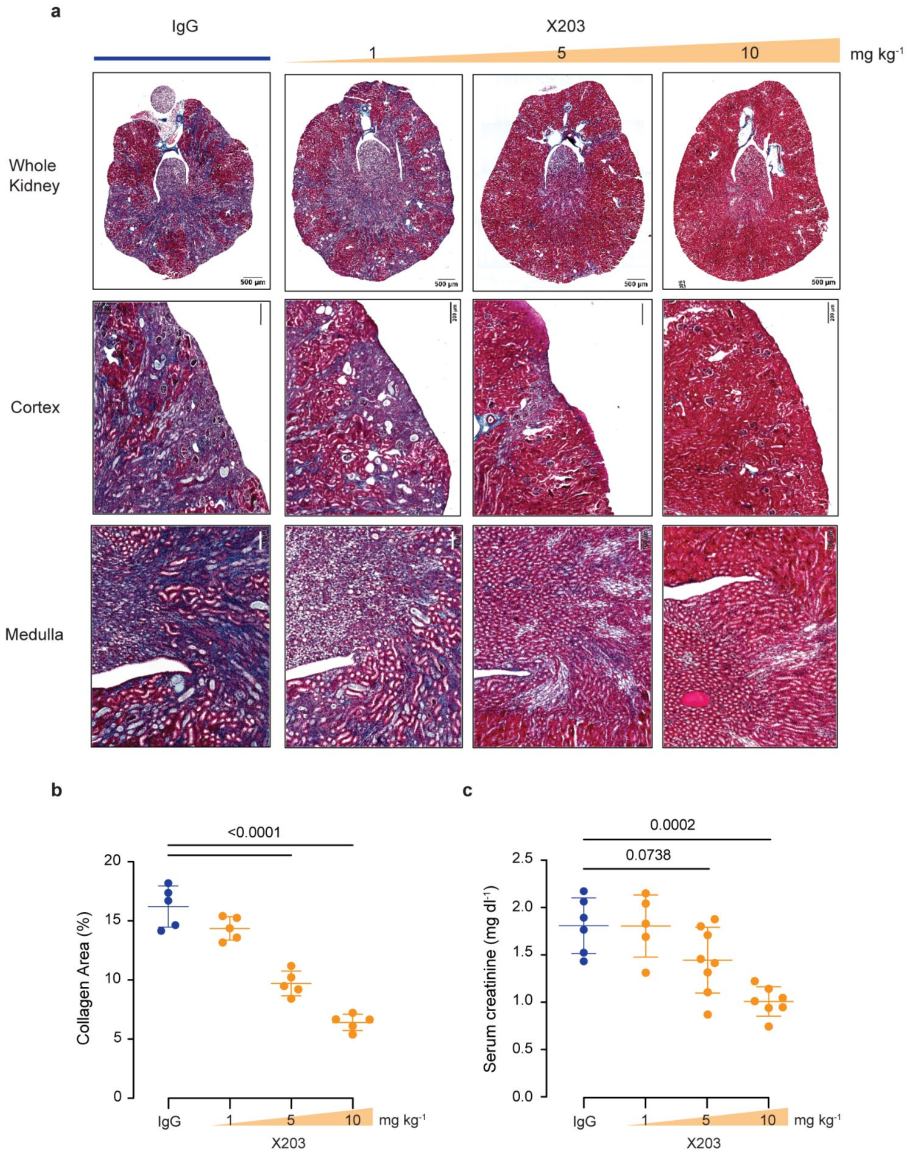

Supplementary Figure 3: Dose-dependent effects of anti-IL11 on renal fibrosis and function. a Representative Masson's Trichrome images of whole kidney cross section (scale bars: 500 µm), kidney cortex (scale bars: 200 µm), and kidney medulla (scale bars: 100 µm) (representative dataset from n=5/group), and **b** quantification of collagen area from Masson's Trichrome-stained kidney sections (n=5/group), **c** serum creatinine for X203 dose finding experiments, as compared to IgG (10 mg/kg), as represented in Schematic Fig. 3a (IgG (n=6), X203 1 mg/kg (n=5), X203 5 mg/kg (n=8), X203 10 mg/kg (n=7)). **b**, **c** Data are shown as mean±SD, one-way ANOVA with Dunnett's correction. Source data are provided as a Source Data file.

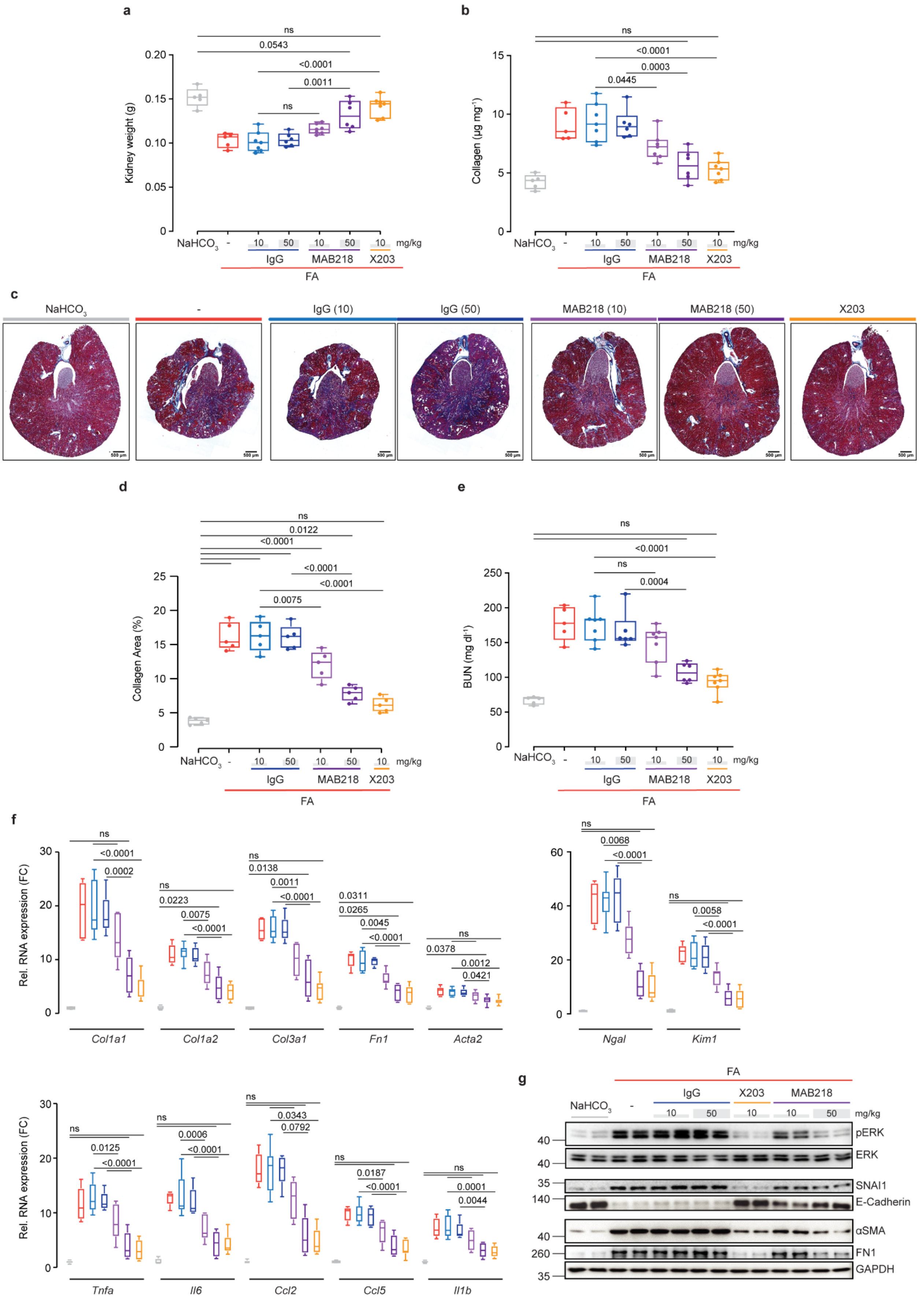

a
b
c
d
e
f
g
Kidney weight (g)
Collagen (μg mg⁻¹)
Collagen Area (%)
BUN (mg dl⁻¹)
Rel. RNA expression (FC)
NaHCO3
IgG
MAB218
X203
FA
mg/kg
IgG (10)
IgG (50)
MAB218 (10)
MAB218 (50)
Col1a1
Col1a2
Col3a1
Fn1
Acta2
Ngal
Kim1
Tnfa
Il6
Ccl2
Ccl5
Il1b
pERK
ERK
SNAI1
E-Cadherin
αSMA
FN1
GAPDH

Supplementary Figure 4: Therapeutic effects of two separate IL11 neutralizing antibodies (X203 and MAB218) on renal phenotypes following folic acid-induced nephrotoxicity. a Kidney weight, **b** kidney collagen content by hydroxyproline assay, **c** representative Masson's Trichrome images of whole kidney cross section (scale bars: 500 μm, representative dataset from n=5/group), **d** quantification of collagen area from Masson's Trichrome-stained kidney sections (n=5/group), **e** BUN, **f** relative renal mRNA expression of *Col1a1*, *Col1a2*, *Col3a1*, *Fn1*, *Acta2, Ngal, Kim1*,*Tnfα*, *Il6, Ccl2*, *Ccl5*, and *Il1β* (n=5-7/group), and **g** Western blots of pERK, ERK, SNAI1, E-Cadherin, αSMA, Fibronectin, and GAPDH (representative dataset from n=5/group) for X203 and MAB218 therapeutic comparison experiments as shown in Schematic Fig. 3d. **a, b, e, f** $NaHC0_3$, FA (n=5/group), IgG/MAB218/X203 (10 mg/kg) (n=7/group), IgG/MAB218 (50 mg/kg) (n=6/group). **a, b, d-f** Data are shown as box-and-whisker with median (middle line), 25th–75th percentiles (box), and minimum-maximum values (whiskers), one-way ANOVA with Tukey's correction except for (**f**, *Ccl2*) which was analyzed by Kruskal-Wallis with Dunn's correction. Source data are provided as a Source Data file.

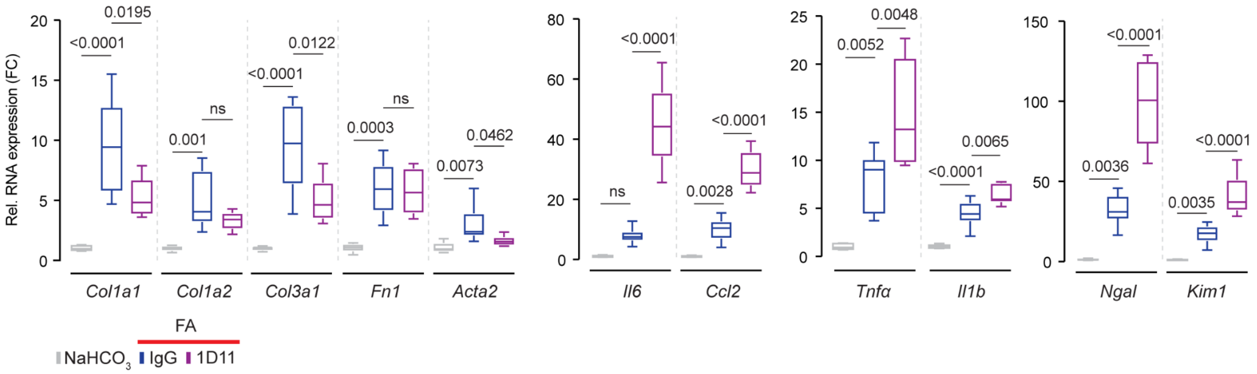

Supplementary Figure 5: Markers of fibrosis, inflammation, and kidney damage in injured kidneys of mice receiving anti-TGFβ (1D11) treatment. Relative renal mRNA expression levels of fibrosis (*Col1a1, Col1a2, Col3a1, Fn1, Acta2),* pro-inflammatory (*Tnfα*, *Il6, Ccl2*, *Il1β*), and kidney injury (*Ngal*, *Kim1*) markers for experiments illustrated in Schematic Fig. 3f ($NaHC0_3$ (n=4), FA+IgG (n=10), FA+1D11 (n=6). Data are shown as box-and-whisker with median (middle line), 25th–75th percentiles (box), and minimum-maximum values (whiskers), one-way ANOVA with Tukey's correction. Source data are provided as a Source Data file.

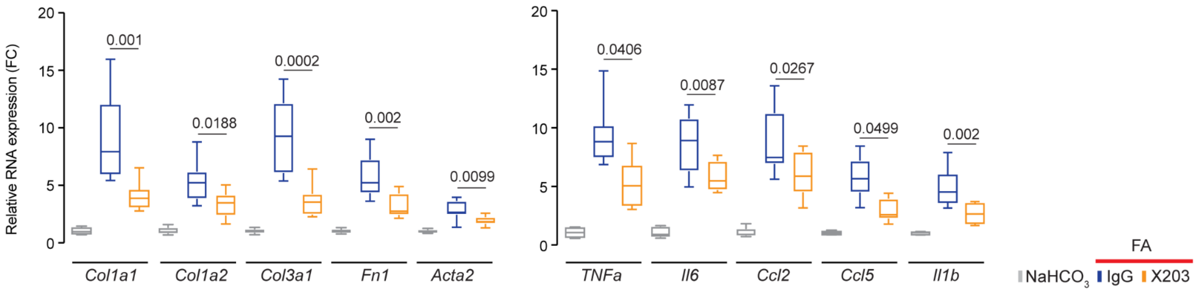

Supplementary Figure 6: Markers of fibrosis and inflammation in the kidneys of mice subjected to folic acid induced renal injury and given X203 in a treatment mode. Relative renal mRNA expression of *Tnfα*, *Il6, Ccl2*, *Ccl5*, *Il1β*, *Col1a1*, *Col1a2*, *Col3a1*, *Fn1*, and *Acta2* for X203 therapeutic dosing experiments as illustrated in Schematic Fig. 4a (NaHC0$_3$ (n=5), FA+IgG (n=9), FA+X203 (n=8)). Data are shown as box-and-whisker with median (middle line), 25th–75th percentiles (box), and minimum-maximum values (whiskers), one-way ANOVA with Tukey's correction except for (*Tnfα*, *Ccl5*) which were analyzed by Kruskal-Wallis with Dunn's correction. Source data are provided as a Source Data file.

.

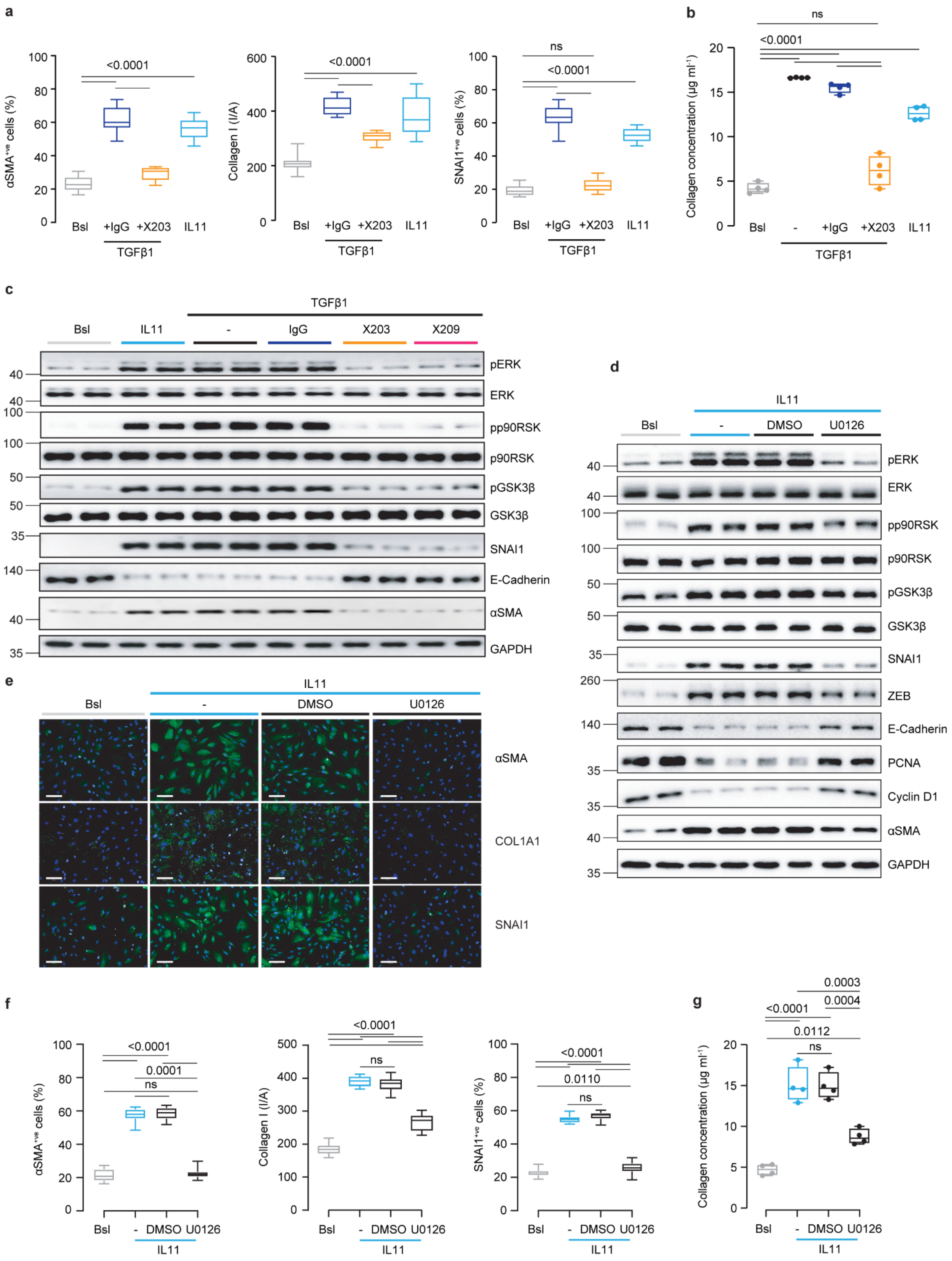

a
αSMA+ve cells (%)
<0.0001
Bsl
+IgG
+X203
IL11
TGFβ1
Collagen I (I/A)
SNAI1+ve cells (%)
ns
b
Collagen concentration (µg ml-1)
c
pERK
ERK
pp90RSK
p90RSK
pGSK3β
GSK3β
SNAI1
E-Cadherin
αSMA
GAPDH
IgG
X203
X209
d
DMSO
U0126
ZEB
PCNA
Cyclin D1
e
COL1A1
f
0.0001
0.0110
g
0.0003
0.0004
0.0112

Supplementary Figure 7: X203 inhibits TEC mesenchymal transition in an ERK dependent manner. **a** Quantification of αSMA^{+ve} and SNAI1^{+ve} cells and Collagen 1 immunostaining (n=14/group) and **b** collagen secretion in the supernatant (n=4/group) from TECs stimulated with either IL11 or TGFβ in the presence of IgG/X203. **c** Western blots of pERK, ERK, pp90RSK, p90RSK, pGSK3β, GSK3β, SNAI1, E-Cadherin, and GAPDH for TECs stimulated with either IL11 or TGFβ in the presence of IgG, X203 (anti-IL11), or X209 (anti-IL11RA) (representative dataset from n=4/group). **d** Western blots of pERK, ERK, pp90RSK, p90RSK, pGSK3β, GSK3β, SNAI1, ZEB, E-Cadherin, PCNA, Cyclin D1, αSMA, and GAPDH (representative dataset from n=4/group), **e** representative IF images (scale bars: 100 μm, representative dataset from n=3/group) and **f** quantification of αSMA^{+ve} and SNAI1^{+ve} cells (n=14/group) and Collagen 1 immunostaining (n=21/group) for TECs stimulated with IL11 alone or in the presence of either DMSO or U0126. **g** Collagen secretion in the supernatant (n=4/group) from bsl, IL11, IL11+DMSO, and IL 11+U0126 group. **a-g** IL11 (5 ng/ml), TGFβ1 (5 ng/ml), IgG/X203/X209 (2 μg/ml), U0126 (10 μM); 24-hour stimulation. **a, b, f, g** Data are shown as box-and-whisker with median (middle line), 25th–75th percentiles (box), and minimum-maximum values (whiskers), one-way ANOVA with Tukey's correction (except for F, αSMA^{+ve} which was analyzed by Kruskal-Wallis with Dunn's correction). Bsl: Baseline; I/A: Intensity/Area. Source data are provided as a Source Data file.

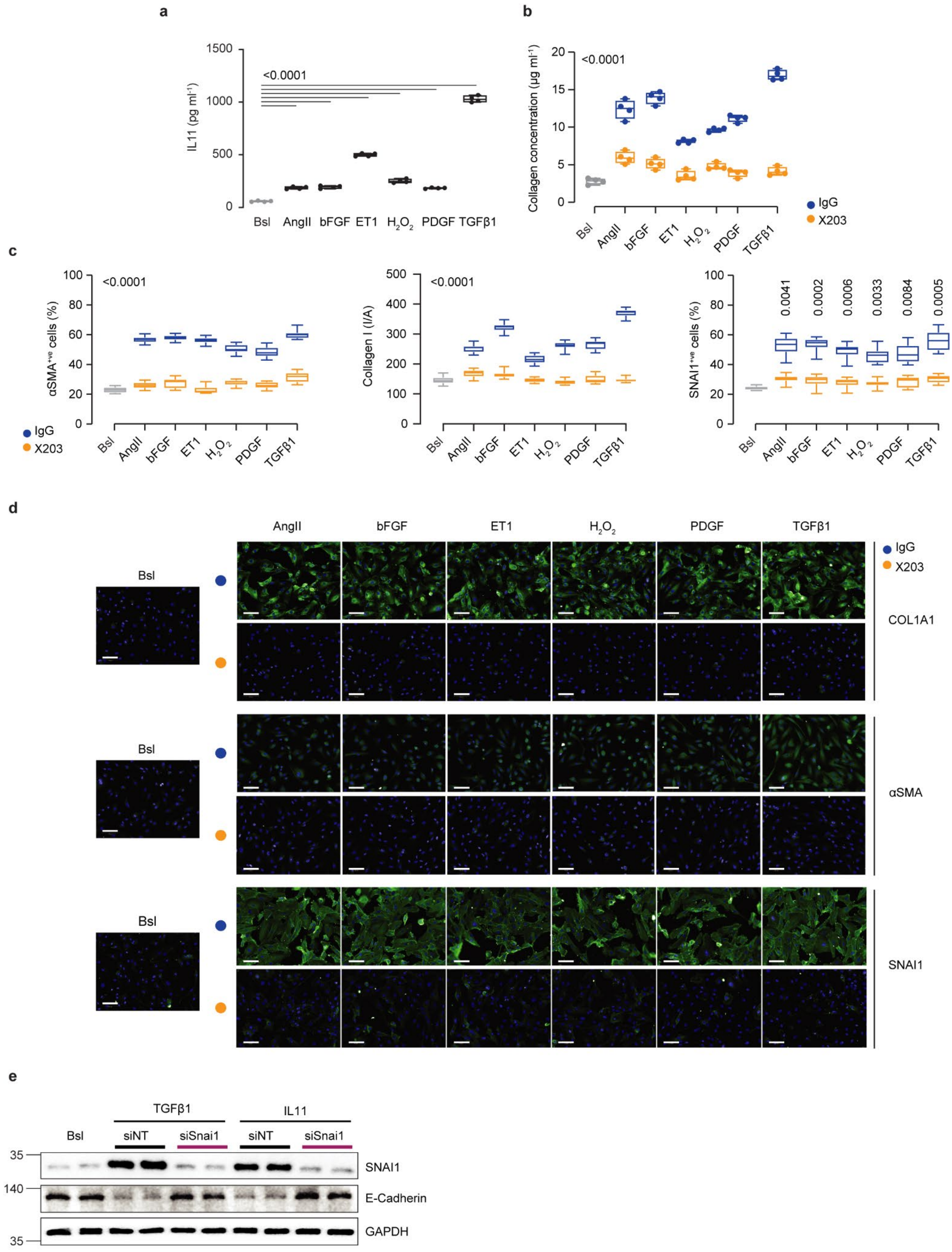

a
IL11 (pg ml⁻¹)
<0.0001
Bsl AngII bFGF ET1 H2O2 PDGF TGFβ1
b
Collagen concentration (µg ml⁻¹)
<0.0001
IgG
X203
c
αSMA+ve cells (%)
Collagen I (I/A)
SNAI1+ve cells (%)
<0.0001
0.0041 0.0002 0.0006 0.0033 0.0084 0.0005
d
AngII bFGF ET1 H2O2 PDGF TGFβ1
Bsl
COL1A1
αSMA
SNAI1
e
TGFβ1
IL11
Bsl
siNT
siSnai1
SNAI1
E-Cadherin
GAPDH
35
140
35

Supplementary Figure 8: IL11 signaling is required for induction of pEMT in TECs. **a** ELISA of secreted IL11 (n=4/group), **b** the amount of secreted collagen in the supernatant (n=4/group), **c** quantification (n=14/group), and **d** representative IF images (scale bars: 100 μm, representative dataset from n=3/group) of αSMA^{+ve} and $SNAI1^{+ve}$ cells and Collagen 1 immunostaining from TECs after 24-hour treatment with Angiontensin II (AngII), basic FGF (bFGF), Endothelin-1 (ET-1), H_20_2, PDGF, and TGFβ. **e** Western blots of SNAI1, E-Cadherin, and GAPDH from TECs stimulated with TGFβ or IL11 subjected to siRNA knockdown for SNAI1 (NT: non targeting siRNA control) (representative dataset from n=4/group). **a-e** TGFβ1 (5 ng/ml), IL11 (5 ng/ml), AngII (100 nM), bFGF (10 ng/ml), Endothelin 1 (ET-1, 250 ng/ml), H_20_2 (0.2 mM), PDGF (20 ng/ml), IgG/X203/(2 μg/ml), siNT/siSNAI1 (25 nM); 24-hour stimulation. **a-c** Data are shown as box-and-whisker with median (middle line), 25th–75th percentiles (box), and minimum-maximum values (whiskers). **a** One-way ANOVA with Dunnett's correction; **b,c** one-way ANOVA with Tukey's correction (except for C, $SNAI1^{+ve}$ which was analyzed by Kruskal-Wallis with Dunn's correction). Bsl: Baseline; I/A: Intensity/Area. Source data are provided as a Source Data file.

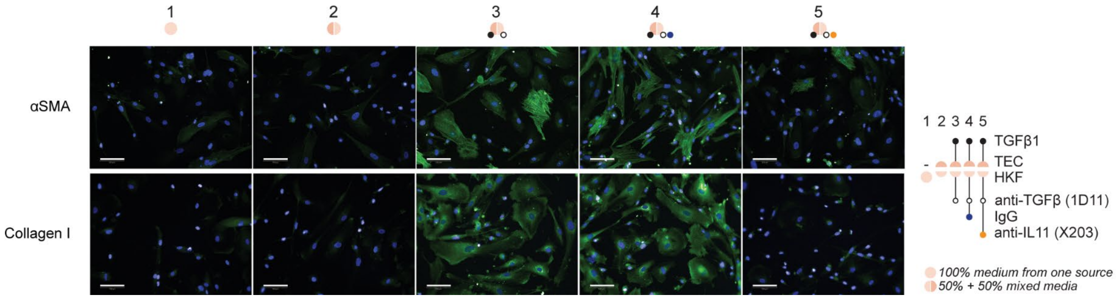

Supplementary Figure 9: Paracrine IL11 (but not TGFβ) activity from TECs causes renal fibroblast-to-myofibroblast transition. Representative images (scale bars: 100 µm, representative dataset from n=3/group) of αSMA^{+ve} cells and Collagen 1 immunostaining from media transfer experiments in which conditioned media from control or TGFβ-stimulated TECs (24 hours) were used to treat primary human kidney fibroblasts (HKFs) in the presence of anti-TGFβ (1D11) alone or with either IgG or X203 as shown by the schematic on the right. Source data are provided as a Source Data file.

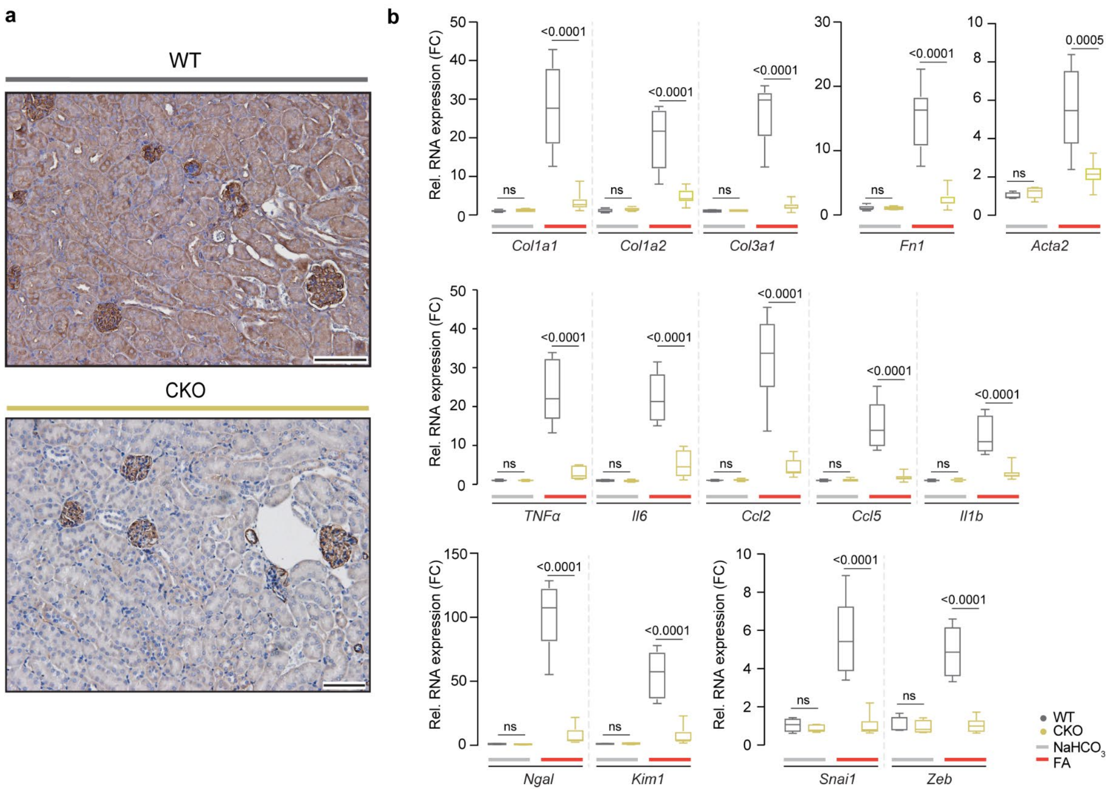

Supplementary Figure 10: Mice with TEC-specific *Il11ra1* deletion are protected from fibrosis, inflammation and tubule damage following AKI. a Representative immunohistochemistry images of IL11RA staining in kidneys from wild-type (WT) and TEC-specific *Il11ra1*-deleted (CKO) mice (scale bars: 100 µm; representative dataset from n=3/group). **b** Relative renal mRNA expression levels of fibrotic markers (*Col1a1, Col1a2, Col3a1, Fn1, Acta2),* kidney injury markers (*Ngal*, *Kim1*), pro-inflammatory markers (*Tnfα*, *Il6, Ccl2*, *Ccl5, Il1β*), and pEMT markers (*Snai1*, *Zeb*) for experiments shown in Schematic Fig. 7a (WT-$NaHC0_3$ (n=4), CKO-$NaHC0_3$ (n=5), WT-FA (n=8), CKO-FA (n=7)). Data are shown as box-and-whisker with median (middle line), 25th–75th percentiles (box), and minimum-maximum values (whiskers), 2-way ANOVA with Sidak's correction. Source data are provided as a Source Data file.

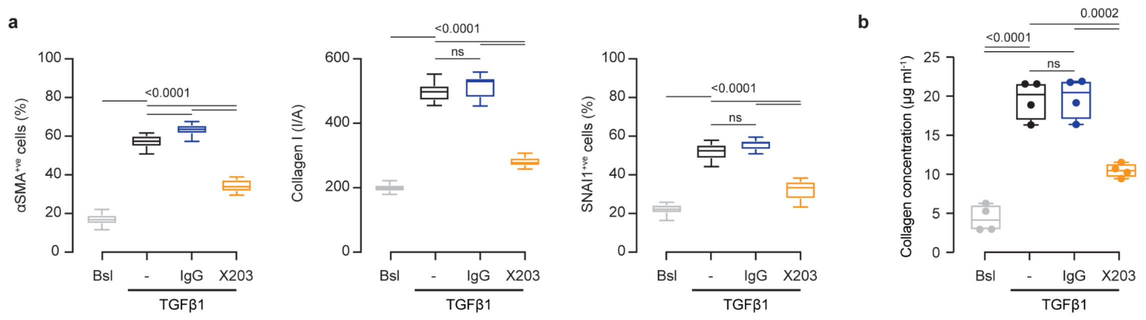

Supplementary Figure 11: Inhibition of IL11 signaling reverts TECs that were in mesenchymal state back to an epithelial phenotype. Quantification of **a** αSMA^{+ve} and SNAI1^{+ve} cells and Collagen 1 immunostaining (n=14/group), and **b** collagen secretion in the supernatant from TEC reversal experiments as shown in Schematic Fig. 6a. Data are shown as box-and-whisker with median (middle line), 25th–75th percentiles (box), and minimum-maximum values (whiskers), one-way ANOVA with Tukey's correction. Source data are provided as a Source Data file.

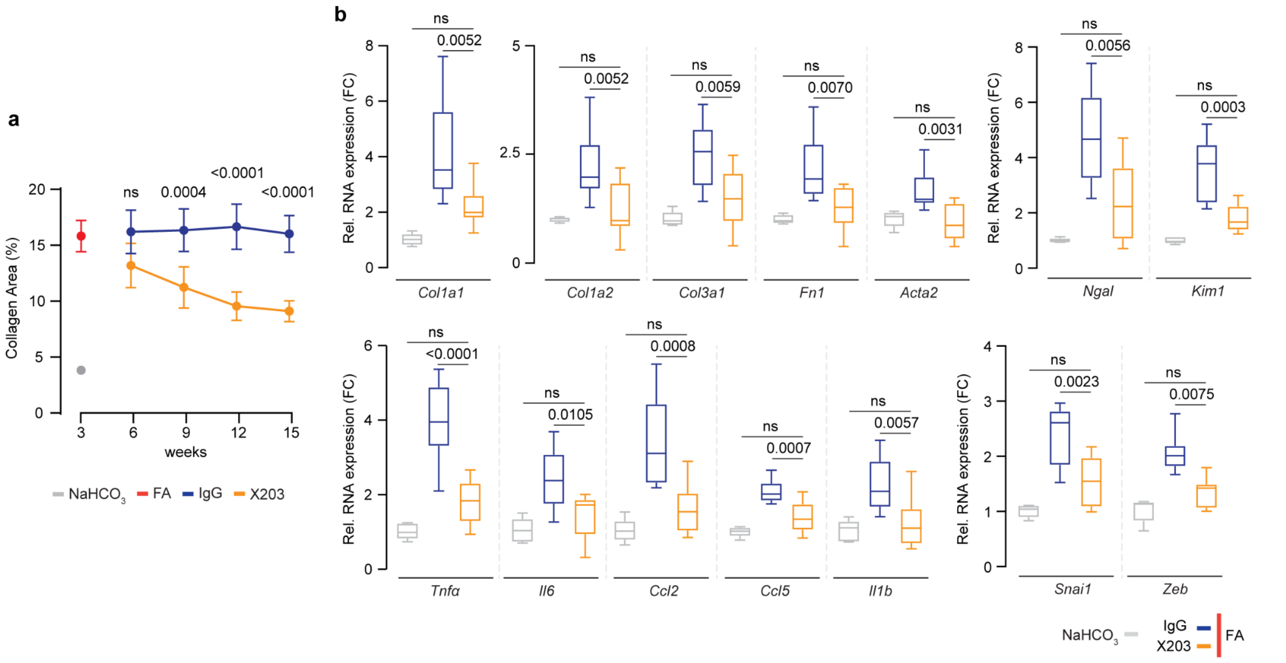

Supplementary Figure 12: X203 reverses pEMT-driven renal fibrosis, inflammation, and parenchymal damage in mice with chronic kidney disease. a Quantification of collagen area from Masson's Trichrome-stained kidney sections (n=4/group for kidneys collected at week 6, 9, and , n=5/group from those collected at other time points). Data are shown as mean ± SD; 2-way ANOVA with Sidak's correction. **b** Relative renal mRNA expression levels of fibrotic markers (*Col1a1, Col1a2, Col3a1, Fn1, Acta2),* kidney injury markers (*Ngal*, *Kim1*), pro-inflammatory markers (*Tnfα*, *Il6, Ccl2*, *Ccl5, Il1β*), and pEMT markers (*Snai1*, *Zeb*) for experiments shown in Schematic Fig. 9a (NaHC0₃ (n=5), FA FA+IgG/X203 (W15) (n=9/group)). Data are shown as box-and-whisker with median (middle line), 25th–75th percentiles (box), and minimum-maximum values (whiskers), one-way ANOVA with Tukey's correction (except for *Zeb* which was analyzed by Kruskal-Wallis with Dunn's correction). Source data are provided as a Source Data file.

Supplementary Table 1: SYBR primer sequences for qPCR

Host	Gene	Forward (5’-3’)	Reverse (5’-3’)
Mouse	*Acta2*	TCCATCGTCCACCGCAAAT	GCCAGGGCTACAAGTTAAGG
	Ccl2	GAAGGAATGGGTCCAGACAT	ACGGGTCAACTTCACATTCA
	Ccl5	GCTGCTTTGCCTACCTCTCC	TCGAGTGACAAACACGACTGC
	Col1a1	GAGCAGACGGGAGTTTCTCCT	CATGTAGACTCTTTGCGGCTG
	Col1a2	AGGATTGGTCAGAGCAGTGT	TCCACAACAGGTGTCAGGGT
	Col3a1	CCACCCAATACAGGTCAAATGC	TGAGTATGACCGTTGCTCTGC
	Gapdh	CTGGAAAGCTGTGGCGTGAT	GACGGACACATTGGGGGTAG
	Il1β	CACAGCAGCACATCAACAAG	GTGCTCATGTCCTCATCCTG
	Kim1	AAACCAGAGATTCCCACACG	GTCGTGGGTCTTCCTGTAGC
	Ngal	TGGCCCTGAGTGTCATGTG	CTCTTGTAGCTCATAGATGGTG C
	Tnfα	ATGAGAAGTTCCCAAATGGC	CTCCACTTGGTGGTTTGCTA
	Snai1	CACACGCTGCCTTGTGTCT	GGTCAGCAAAAGCACGGTT
	Zeb	GCTGGCAAGACAACGTGAAAG	GCCTCAGGATAAATGACGGC
Human	*DUSP5*	GCCAGCTTATGACCAGGGTG	GTCCGTCGGGAGACATTCAG

Uncropped blots for supplementary figures

Supplementary Figure 4g

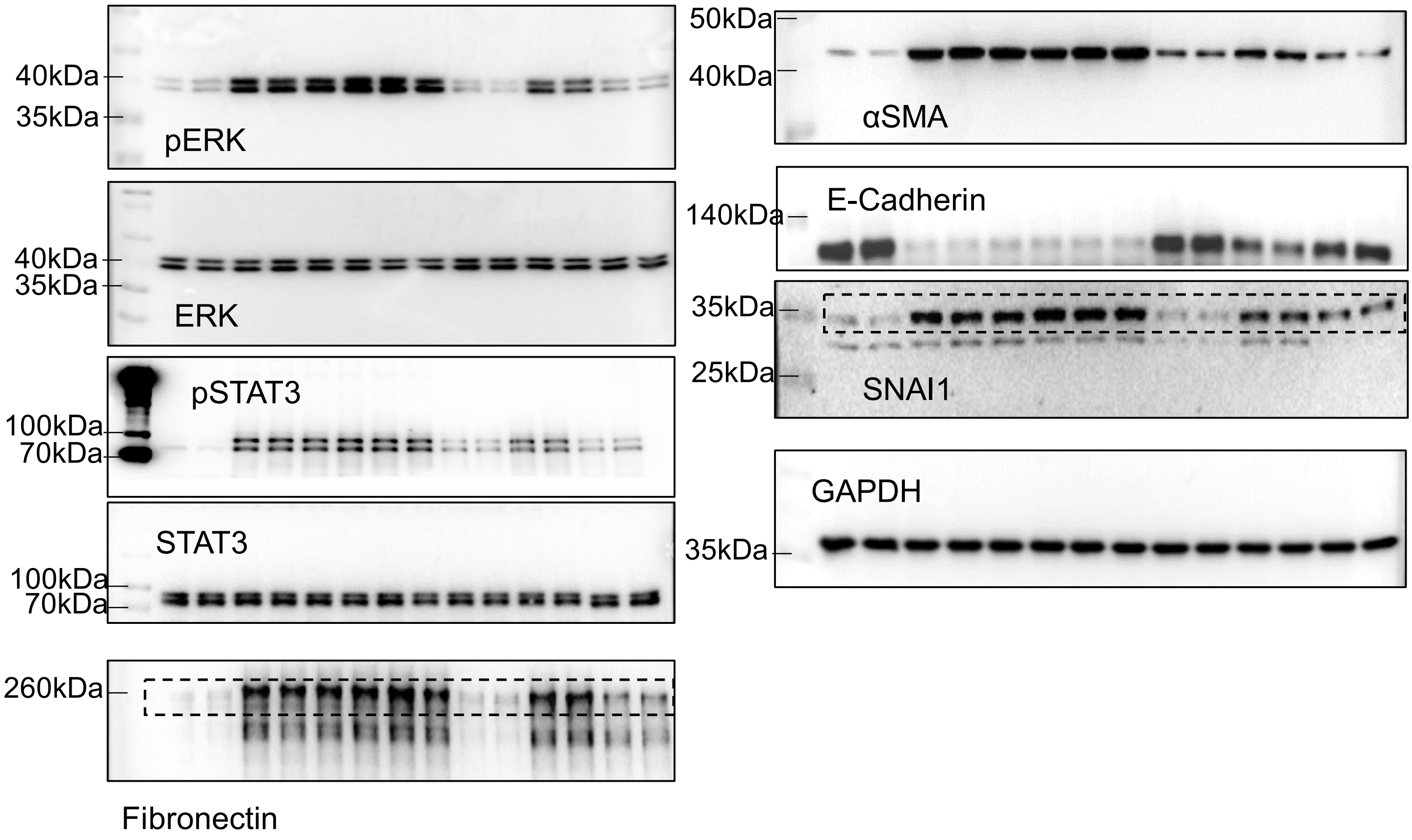

Supplementary Figure 7c

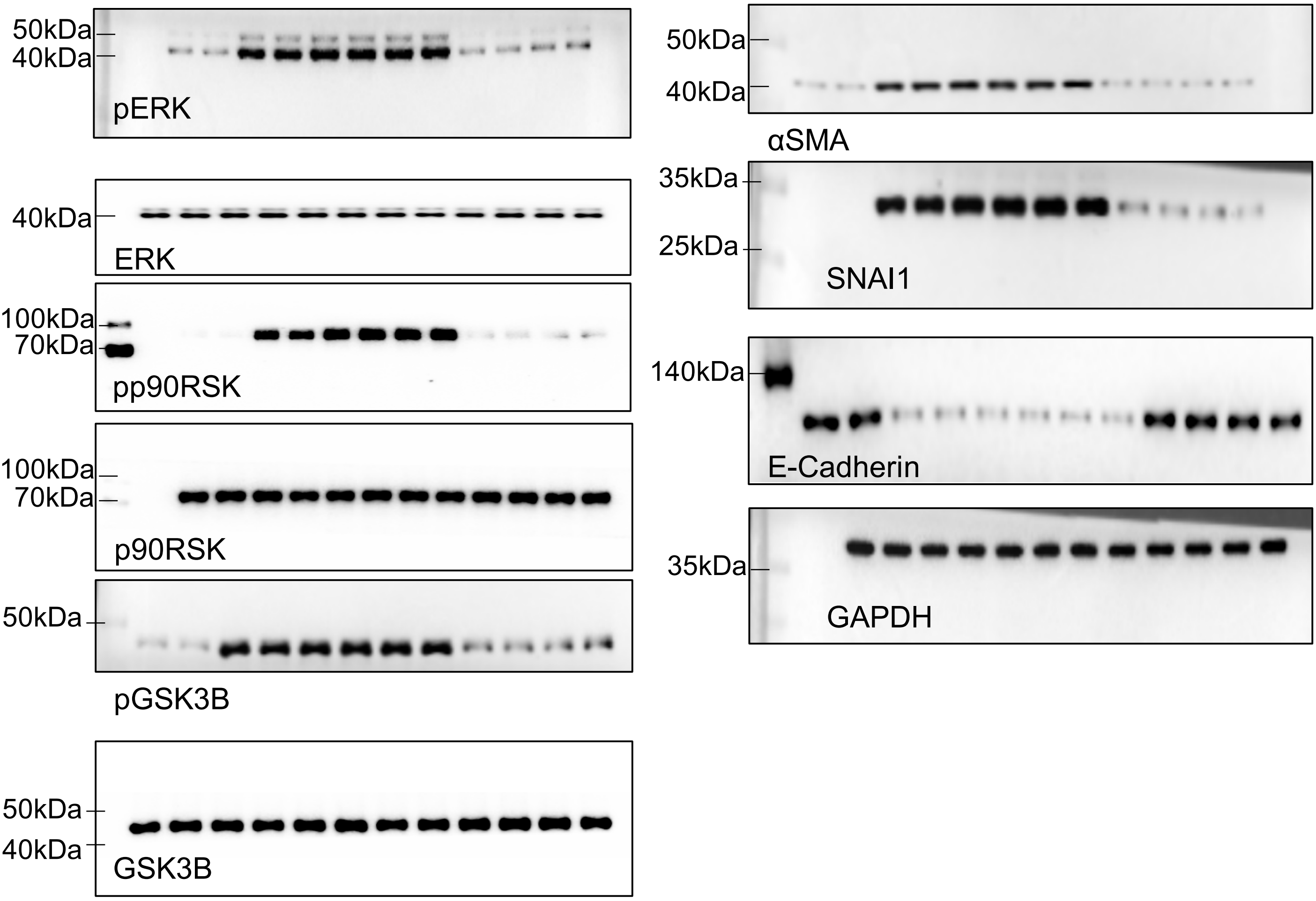

Supplementary Figure 7d

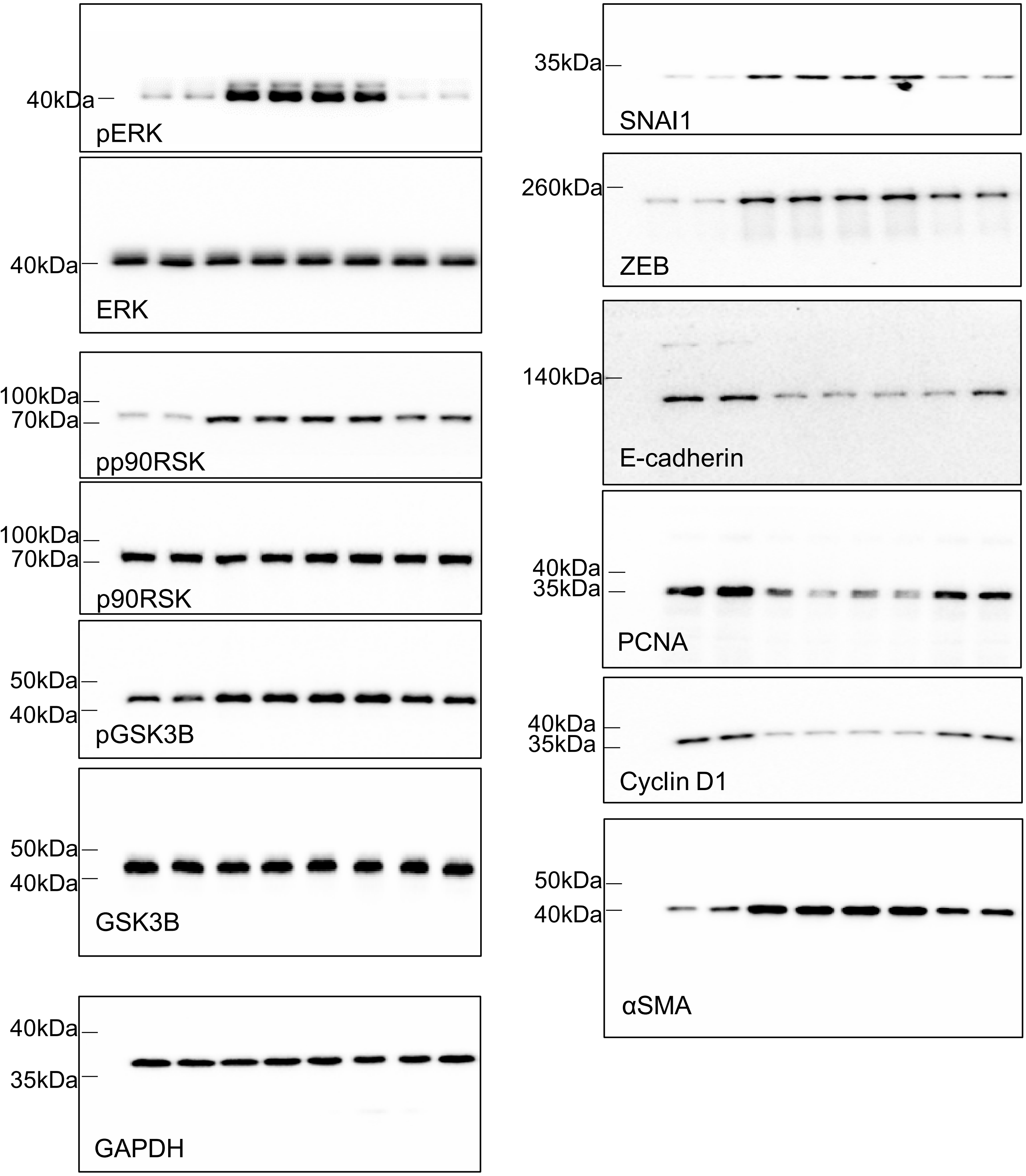

Supplementary 8e

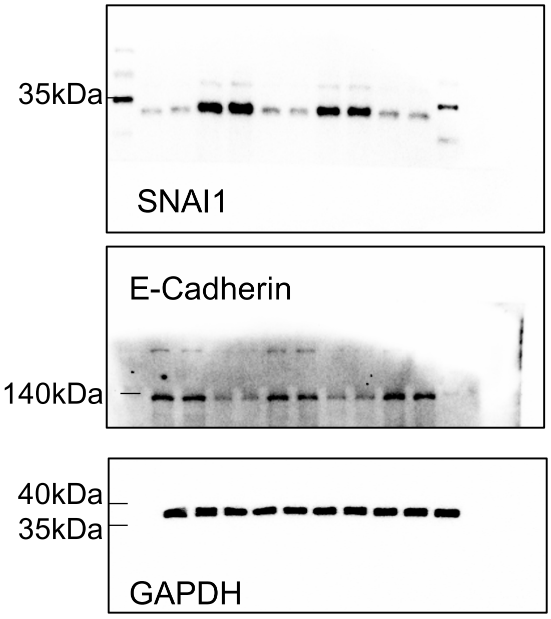

Supplement: Supplementary file 1 — Supplementary Information [file 41467_2022_35306_MOESM1_ESM.pdf]
