# Supplementary material for: Targeting endogenous kidney regeneration using anti-IL11 therapy in acute and chronic models of kidney disease
Source: Nat Commun. 2022 Dec 5;13:7497. doi: 10.1038/s41467-022-35306-1 (PMC9723120; doi:10.1038/s41467-022-35306-1)
Supplement: Supplementary file 4 — Reporting Summary [file 41467_2022_35306_MOESM4_ESM.pdf]

nature research

Corresponding author(s): Anissa A Widjaja and Stuart A Cook

Last updated by author(s): Nov 14, 2022

Reporting Summary

Nature Research wishes to improve the reproducibility of the work that we publish. This form provides structure for consistency and transparency in reporting. For further information on Nature Research policies, see our Editorial Policies and the Editorial Policy Checklist.

Statistics

For all statistical analyses, confirm that the following items are present in the figure legend, table legend, main text, or Methods section.

n/a	Confirmed	
☐	☒	The exact sample size (n) for each experimental group/condition, given as a discrete number and unit of measurement
☐	☒	A statement on whether measurements were taken from distinct samples or whether the same sample was measured repeatedly
☐	☒	The statistical test(s) used AND whether they are one- or two-sided<br>*Only common tests should be described solely by name; describe more complex techniques in the Methods section.*
☒	☐	A description of all covariates tested
☐	☒	A description of any assumptions or corrections, such as tests of normality and adjustment for multiple comparisons
☐	☒	A full description of the statistical parameters including central tendency (e.g. means) or other basic estimates (e.g. regression coefficient) AND variation (e.g. standard deviation) or associated estimates of uncertainty (e.g. confidence intervals)
☐	☒	For null hypothesis testing, the test statistic (e.g. F, t, r) with confidence intervals, effect sizes, degrees of freedom and P value noted<br>*Give P values as exact values whenever suitable.*
☒	☐	For Bayesian analysis, information on the choice of priors and Markov chain Monte Carlo settings
☒	☐	For hierarchical and complex designs, identification of the appropriate level for tests and full reporting of outcomes
☒	☐	Estimates of effect sizes (e.g. Cohen's d, Pearson's r), indicating how they were calculated

Our web collection on statistics for biologists contains articles on many of the points above.

Software and code

Policy information about availability of computer code

Data collection	The study did not involve software and code for data collection.
Data analysis	GraphPad Prism (version 9.4.1), Harmony software (version 3.5.2), ImageJ (version 1.53f51)

For manuscripts utilizing custom algorithms or software that are central to the research but not yet described in published literature, software must be made available to editors and reviewers. We strongly encourage code deposition in a community repository (e.g. GitHub). See the Nature Research guidelines for submitting code & software for further information.

Data

Policy information about availability of data

All manuscripts must include a data availability statement. This statement should provide the following information, where applicable:

- Accession codes, unique identifiers, or web links for publicly available datasets
- A list of figures that have associated raw data
- A description of any restrictions on data availability

All data are available within the Article or Supplementary Information. The RNA-seq data reported in this study have been deposited in the GEO database under the NCBI GEO accession number: GSE199080 (https://www.ncbi.nlm.nih.gov/geo/query/acc.cgi?acc=GSE199080).Source data are provided with this paper: raw data are provided in Source Data file 1, densitometry analysis is provided in Source Data file 2, and uncropped blots for main figures are provided in Source Data file 3.

Field-specific reporting

Please select the one below that is the best fit for your research. If you are not sure, read the appropriate sections before making your selection.

- [x] Life sciences
- [ ] Behavioural & social sciences
- [ ] Ecological, evolutionary & environmental sciences

For a reference copy of the document with all sections, see nature.com/documents/nr-reporting-summary-flat.pdf

Life sciences study design

All studies must disclose on these points even when the disclosure is negative.

Sample size	Sample sizes were based on the experience of the authors with molecular and invivo studies as published in many studies.<br>For animal models, experiments were designed to detect differences between treatment groups or genotype-dependent effects at 80% power (α=0.05).<br>Sample sizes may vary depending on animal availability<br>Sample size for cell based assays were determined based on sample availability.
Data exclusions	No data was excluded from the analyses.
Replication	All experiments were repeated with reproducibility. The replication number for each experiment is indicated in the legend of the corresponding figure.
Randomization	For in vitro studies, equal number of cells were seeded and allocated randomly into experimental group. For in vivo studies, mice were randomly allocated to experimental groups on the day of the treatment to ensure equal litter/age across groups except for Il11 KO and Il11ra CKO mice in which randomization was assigned within the same genotypes.
Blinding	For in vitro experiments, investigators were not blinded to group allocation during data collection and analysis as they were performed by a single individual. For in vivo experiments, treatments/genotypes were not disclosed to investigators generating quantitative readouts during data collection but investigators were not blinded during data analysis as those who did the analysis are the same individuals who did the blinding. Histological analysis were performed blinded to treatments and genotypes.

Reporting for specific materials, systems and methods

We require information from authors about some types of materials, experimental systems and methods used in many studies. Here, indicate whether each material, system or method listed is relevant to your study. If you are not sure if a list item applies to your research, read the appropriate section before selecting a response.

Materials & experimental systems

n/a	Involved in the study	
[ ]	[x]	Antibodies
[ ]	[x]	Eukaryotic cell lines
[x]	[ ]	Palaeontology and archaeology
[ ]	[x]	Animals and other organisms
[x]	[ ]	Human research participants
[x]	[ ]	Clinical data
[x]	[ ]	Dual use research of concern

Methods

n/a	Involved in the study	
[x]	[ ]	ChIP-seq
[x]	[ ]	Flow cytometry
[x]	[ ]	MRI-based neuroimaging

Antibodies

Antibodies used

The following primary antibodies were used for Western Blot. They are listed as antigen followed by catalog number, clone name (for monoclonal antibody), supplier name, and dilution ratio:
1. Cyclin D1, 55506, clone E3P5S, CST, 1:1000.
2. E-Cadherin, 3195, clone 24E10, CST, 1:1000.
3. DUSP5, ab200708, clone EPR19684, abcam, 1:1000.
4. pERK1/2, 4370, clone D13.14.4E, CST, 1:1000.
5. ERK1/2, 4695, clone 137F5, CST, 1:1000.
6. Fibronectin, ab2413, NA, abcam, 1:1000.
7. GAPDH 2118, clone 14C10, CST, 1:1000.
8. pGSK3β, 5558, clone D85E12, CST, 1:1000.
9. GSK3β, 12456, clone D5C5Z, CST, 1:1000.
10. IL11, NA, clone X203, Aldevron, 1:1000.
11. IL11RA, NA, clone X209, Aldevron, 1:1000.

12. pMEK1/2, 9154, clone 41G9, CST, 1:1000.
13. MEK1/2, 4694, clone L38C12, CST, 1:1000.
14. pp90RSK, 11989, clone D3H11, CST, 1:1000.
15. RSK, 9355, clone 32D7, CST, 1:1000.
16. PCNA, 13110, clone D3H8P, CST, 1:1000.
17.SMA, 19245, clone D4K9N, CST, 1:1000.
18. SNAI1, 3879, clone C15D3, CST, 1:1000.
19. pSTAT3, 4113, clone M9C6, CST, 1:1000.
20. STAT3, 4904, clone 79D7, CST, 1:1000.
21. ZEB, 70512, clone E2G6Y, CST, 1:1000.
22. HRP conjugated anti-mouse IgG (H+L)/anti-mouse HRP, 7076, NA, CST, 1:1000.
23. HRP conjugated anti-rabbit IgG (H+L)/anti-rabbit HRP, 7074, NA, CST, 1:1000.

The following primary antibodies were used for neutralization study (in vitro/in vivo treatment). They are listed as antigen followed by catalog number, clone name (for monoclonal antibody), supplier name, and dilution ratio:
1. IgG, NA, clone 11E10, Aldevron, in vitro: 2 µg/ml, in vivo: doses vary between 10-20 mg/kg as outlined in the respective figure legends.
2. IL11, NA, clone X203, Aldevron, in vitro: 2 µg/ml, in vivo: doses vary between 1-20 mg/kg as outlined in the respective figure legends.
3. IL11RA, NA, clone X209, Aldevron, in vitro: 2 µg/ml.
4. TGFβ, NA, clone 1D11, Aldevron, in vitro: 2 µg/ml, in vivo: 20 mg/kg.

The following primary antibodies were used for Operetta phenotyping assay. They are listed as antigen followed by catalog number, clone name (for monoclonal antibody), supplier name, and dilution ratio:
1. Collagen I, ab34710, NA, abcam, 1:500.
2.SMA, ab7817, NA, abcam, 1:500.
3. SNAI1 (ab180714, NA, abcam, 1:500.
4. Anti-rabbit Alexa Fluor 488, ab150077, NA, abcam, 1:1000.
5. Anti-mouse Alexa Fluor 488, ab150113, NA, abcam, 1:1000.

The following primary antibodies were used for immunofluoresence. They are listed as antigen followed by catalog number, clone name (for monoclonal antibody), supplier name, and dilution ratio:
1. GFP, ab6673, NA, abcam, 1:1000.
2. E-Cadherin, 3195, clone 24E10, CST, 1:1000
3. IL11RA, ab125015, NA, abcam, 1:200.
4. Anti-goat Alexa Fluor 488, ab150129, NA, abcam, 1:250.
5. Anti-rabbit Alexa Fluor 488, ab150077, NA, abcam, 1:500.
6. Anti-rabbit Alexa Fluor 647, ab150079, NA, abcam, 1:250.

The following primary antibodies were used for immunohistochemistry. They are listed as antigen followed by catalog number, clone name (for monoclonal antibody), supplier name, and dilution ratio:
1. IL11, NA, clone X203, Aldevron, 1:250.
2. IL11RA, NA, clone X209, Aldevron, 1:250.

Validation

All commercially available antibodies are validated by the manufacturers as indicated on the respective manufacturer's website. The custom-made antibodies i.e: IgG (11E10), anti-IL11 (X203), anti-IL11RA (X209), and anti-TGFB (1D11) were validated by citations. Manufacturer's website containing validation data for the commercially available antibodies and citations for the custom-made antibodies are listed below:
1. Collagen I: Human; IF (https://www.abcam.com/collagen-i-antibody-ab34710.html).
2. Cyclin D1: Human, mouse; WB (https://www.cellsignal.com/products/primary-antibodies/cyclin-d1-e3p5s-xp-rabbit-mab/55506).
3. E-Cadherin: Human, mouse; WB (https://www.cellsignal.com/products/primary-antibodies/e-cadherin-24e10-rabbit-mab/3195).
4. DUSP5: Human, WB (https://www.abcam.com/dusp5-antibody-epr19684-ab200708.html).
5. phospho-ERK1/2: Human, mouse; WB (https://www.cellsignal.com/products/primary-antibodies/phospho-p44-42-mapk-erk1-2-thr202-tyr204-d13-14-4e-xp-rabbit-mab/4370?Ntk=Products&Ntt=4370).
6. ERK1/2: Human, mouse; WB (https://www.cellsignal.com/products/primary-antibodies/p44-42-mapk-erk1-2-137f5-rabbit-mab/4695?Ntk=Products&Ntt=4695).
7. Fibronectin: Mouse; WB (https://www.abcam.com/fibronectin-antibody-ab2413.html).
8. GAPDH: Human, mouse; WB (https://www.cellsignal.com/products/primary-antibodies/gapdh-14c10-rabbit-mab/2118).
9. GFP: Mouse; IF (https://www.abcam.com/gfp-antibody-ab6673.html).
10. phospho-GSK3β: Human, mouse; WB (https://www.cellsignal.com/products/primary-antibodies/phospho-gsk-3b-ser9-d85e12-xp-rabbit-mab/5558).
11. GSK3β: Human, mouse; WB (https://www.cellsignal.com/products/primary-antibodies/gsk-3b-d5c5z-xp-rabbit-mab/12456).
12. IgG (11E10) was validated for its non-ability to have any effect on human/mouse cells in PMID: 34108253.
13. IL11 (X203) was validated for neutralization of human and mouse IL11 in PMID: 31554736, for WB in PMID: 31078624, and for IHC in this manuscript.
14. IL11 (MAB218): Human, mouse; neutralization (https://www.rndsystems.com/products/human-il-11-antibody-22626_mab218).
15. IL11RA (X209) was validated for neutralization of human and mouse IL11RA in PMID: 31078624, 34108253, for WB (in this manuscript), for IHC in PMID: 35140116.
16. IL11RA: Human; IF (https://www.abcam.com/il-11ra-antibody-epr5446-ab125015.html).

17. phospho-MEK1/2: Human; WB (https://www.cellsignal.com/products/primary-antibodies/phospho-mek1-2-ser217-221-41g9-rabbit-mab/9154).
18. MEK1/2: Human; WB (https://www.cellsignal.com/products/primary-antibodies/mek1-2-l38c12-mouse-mab/4694).
19. phospho-p90RSK: Human, mouse; WB (https://www.cellsignal.com/products/primary-antibodies/phospho-p90rsk-ser380-d3h11-rabbit-mab/11989).
20. p90RSK: Human, mouse; WB (https://www.cellsignal.com/products/primary-antibodies/rsk1-rsk2-rsk3-32d7-rabbit-mab/9355).
21. PCNA: Human; WB (https://www.cellsignal.com/products/primary-antibodies/pcna-d3h8p-xp-rabbit-mab/13110).
22. aSMA: Human; IF (https://www.abcam.com/alpha-smooth-muscle-actin-antibody-1a4-ab7817.html).
23. aSMA: Human, mouse; WB (https://www.cellsignal.com/products/primary-antibodies/a-smooth-muscle-actin-d4k9n-xp-rabbit-mab/19245)
24. SNAI1: Human; IF (https://www.abcam.com/snail--slug-antibody-ab180714.html).
25. SNAI1: Human, mosue; WB (https://www.cellsignal.com/products/primary-antibodies/snail-c15d3-rabbit-mab/3879).
26. phospho-STAT3: Human, mouse; WB (https://www.cellsignal.com/products/primary-antibodies/phospho-stat3-tyr705-m9c6-mouse-mab/4113?Ntk=Products&Ntt=4113).
27. STAT3: Human, mouse; WB (https://www.cellsignal.com/products/primary-antibodies/stat3-79d7-rabbit-mab/4904?Ntk=Products&Ntt=4904).
28. TGFβ (1D11): Human, mouse; neutralization (PMID: 2537357, 12538738).
29. ZEB: Human, mouse; WB (https://www.cellsignal.com/products/primary-antibodies/zeb1-e2g6y-xp-rabbit-mab/70512).
30. Anti-rabbit HRP: Human, mouse; WB (https://www.cellsignal.com/products/secondary-antibodies/anti-rabbit-igg-hrp-linked-antibody/7074).
31. Anti-moue HRP: Human, mouse; WB (https://www.cellsignal.com/products/secondary-antibodies/anti-mouse-igg-hrp-linked-antibody/7076).
32. Anti-rabbit Alexa Fluor 488: IF (https://www.abcam.com/goat-rabbit-igg-hl-alexa-fluor-488-ab150077.html).
33. Anti-rabbit Alexa Fluor 647: IF (https://www.abcam.com/goat-rabbit-igg-hl-alexa-fluor-647-ab150079.html).
34. Anti-mouse Alexa Fluor 488: IF (https://www.abcam.com/goat-mouse-igg-hl-alexa-fluor-488-ab150113.html).
35. Anti-goat Alexa Fluor 488: IF (https://www.abcam.com/donkey-goat-igg-hl-alexa-fluor-488-ab150129.html).

Eukaryotic cell lines

Policy information about cell lines

Cell line source(s)	Primary human renal proximal tubular epithelial cells (TECs, #4100, lot #19754, ScienCell) isolated from a healthy human kidney (20-year-old female).<br>Primary human kidney fibroblasts (P10666, Lot 20115ty, InnoProt) isolated from a healthy human kidney (59 year old male)
Authentication	Primary human renal proximal tubular epithelial cells were authenticated by ScienCell.<br>Primary human Kidney Fibroblasts were authenticated by InnoProt.
Mycoplasma contamination	All cell lines were tested to be free of mycoplasma contamination.
Commonly misidentified lines (See ICLAC register)	No commonly misidentified cell lines were used in the study.

Animals and other organisms

Policy information about studies involving animals; ARRIVE guidelines recommended for reporting animal research

Laboratory animals

Animal models
Mice were housed in temperatures of 21–24°C with 40–70% humidity on a 12h light/12h dark cycle and provided with food and water ad libitum. For mouse model of folic acid (FA)-induced acute kidney injury (AKI), AKI was induced by intraperitoneal (IP) injection of folic acid (FA, 200 mg/kg) in vehicle (0.3M $NaHCO_3$) to 10-12-week old male mice; control mice were administered vehicle alone. Unilateral ureteral obstruction (UUO) surgeries were carried out on 12-13-week old male mice to generate a mouse model accelerated chronic kidney disease. Briefly, mice were anesthetized by IP injection of ketamine (100 mg/kg) /xylazine (10 mg/kg) and full depth of anaesthesia was accessed with the pedal reflex. Mice were then shaved on the left side of the abdomen. A vertical incision was made through the skin with a scalpel, a second incision was made through the peritoneum to reveal the kidney. Using forceps, the left kidney was brought to the surface and the ureter was tied with surgical silk, twice, below the kidney. The ligated kidney was placed back into its correct anatomical position and sterile saline was added to replenish the loss of fluid. The incisions were then sutured. Animals were post-operatively treated with antibiotic enrofloxacin (15 mg/kg, SC) and analgesic buprenorphine (0.1 mg/kg, SC) for three consecutive days. Mice were sacrificed 9 days post UUO (D10). The number of mice used in each experiment is outlined in the respective figure legends

In vivo administration of anti-IL11 (X203 or MAB218) or anti-TGFβ
10-13-week old C57BL/6J male mice (InVivos, Singapore (SG)) were administered anti-IL11 (X203 or MAB218), anti-TGFβ (1D11), or IgG isotype control (11E10) by intraperitoneal injection at different times, doses (1-20 mg/kg), and durations depending on the experiments as outlined in the main text, figures, and/or figure legends.

Il11-EGFP mice
Transgenic mice with EGFP constitutively knocked-in to the Il11 gene were generated by Cyagen Biosciences Inc21. For FA experiments, 10-12-week old male Il11EGFP/+ mice were IP injected with 150 mg/kg of FA in vehicle (0.3M $NaHCO_3$), aged-matched Il11EGFP/+ littermates receiving equal volume of vehicle were used as controls. Mice were sacrificed at different time points as outlined in the main text, figures, and/or figure legends. For UUO experiments, 12-13 week old male Il11EGFP/+ mice were subjected

to UUO surgery as described above; contralateral (right) kidneys were used as controls.

Tubular epithelial cells (TEC)-specific Il11ra1-deleted mice
To induce the specific deletion of Il11ra1, we crossed Ksp1.3/Cre transgenic mice (B6.Cg-Tg(Cdh16-cre)91Igr/J, Jackson Laboratory), which express Cre recombinase under the control of Cadherin 16 (Cdh16) promoter, with homozygous Il11ra1-floxed mice. Exons 4 to 7 of the Il11ra1 gene were flanked by loxP sites (Il11ra1loxP/loxP) allowing for deletion of Il11ra1 upon Cre recombinase-mediated excision21. Knockdown efficiency was determined by Western blotting of renal IL11RA expression. 10-12-week old male Cdh16Cre/+Il11ra1loxP/loxP (CKO) and Cdh16-/-Il11ra1loxP/loxP mice, which were used as wild-type (WT) controls, were injected with 200 mg/kg of FA or an equal amount of vehicle.

Urine collection
Two days prior to the end of the study, 24-hour urine collections were obtained from mice that are housed in individual metabolic cages with free access to water and food.

EdU incorporation studies
12-week old male C57BL/6J mice (InVivos, Singapore (SG)) were administered 20 mg/kg (2x/week) of anti-IL11 (X203) or IgG for 6 weeks, starting from day 21 post FA (200 mg/kg) injection. Mice were administered a daily intraperitoneal injection of EdU (20 mg/kg) in sterile, phosphate buffered saline (PBS, pH 7.4) for 5 days before they were sacrificed at week 9 post FA.

Wild animals

The study did not involve wild animals.

Field-collected samples

The study did not involve field collected animals.

Ethics oversight

Animal studies were carried out in compliance with the recommendations in the Guidelines on the Care and Use of Animals for Scientific Purposes of the National Advisory Committee for Laboratory Animal Research (NACLAR). All experimental procedures were approved (SHS/2019/1482 and SHS/2019/1483) and conducted in accordance with the SingHealth Institutional Animal Care and Use Committee (IACUC). All mice were provided food and water ad libitum.

Note that full information on the approval of the study protocol must also be provided in the manuscript.
