# Supplementary material for: Targeting endogenous kidney regeneration using anti-IL11 therapy in acute and chronic models of kidney disease
Source: Nat Commun. 2022 Dec 5;13:7497. doi: 10.1038/s41467-022-35306-1 (PMC9723120; doi:10.1038/s41467-022-35306-1)
Supplement: Supplementary file 6 — Description of Additional Supplementary Files [file 41467_2022_35306_MOESM6_ESM.pdf]

Title: Supplementary Data 1:
Description: List of differentially expressed genes in primary human tubular epithelial cells (TEC) stimulated with IL11 for 1, 6, and 24 h as compared to unstimulated TECs. Differential expression analysis is carried out using Wald test in DESeq2 and p-values adjusted using Benjamini & Hochberg method.

Title: Supplementary Data 2:
Description: Supporting statistical information.
