# Supplementary material for: Targeting endogenous kidney regeneration using anti-IL11 therapy in acute and chronic models of kidney disease
Source: Nat Commun. 2022 Dec 5;13:7497. doi: 10.1038/s41467-022-35306-1 (PMC9723120; doi:10.1038/s41467-022-35306-1)
Supplement: Supplementary file 7 — source data file [file 41467_2022_35306_MOESM7_ESM.zip › Source Data file 3.pdf]

Figure 1d

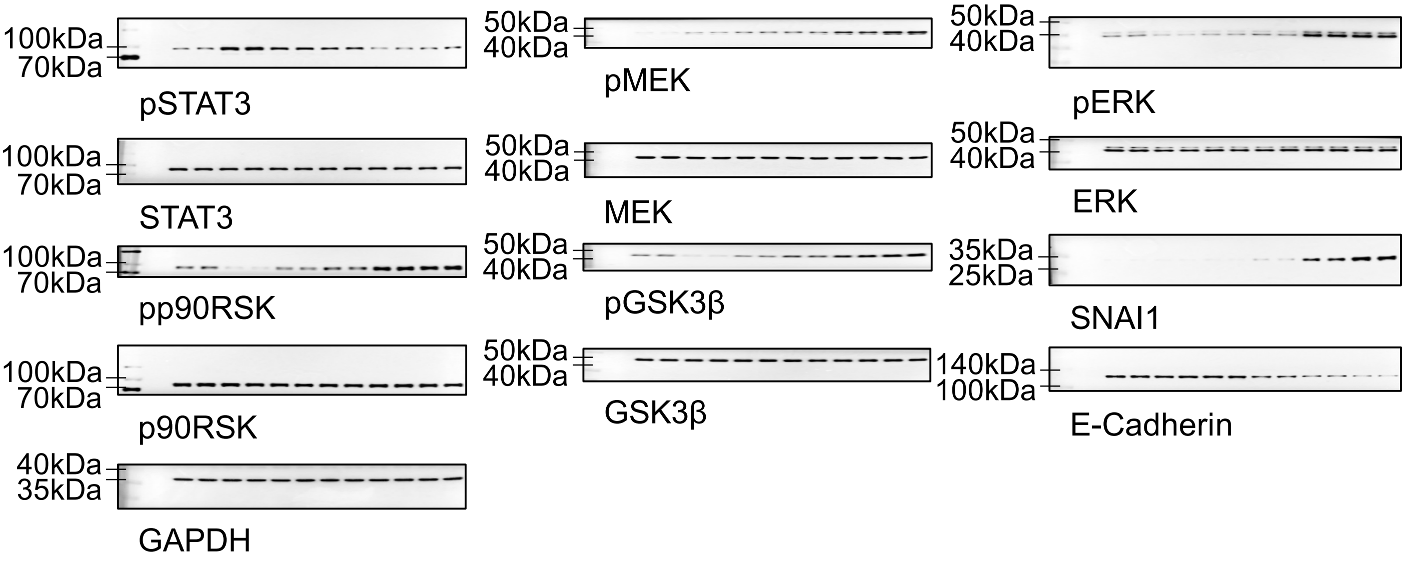

Figure 1h

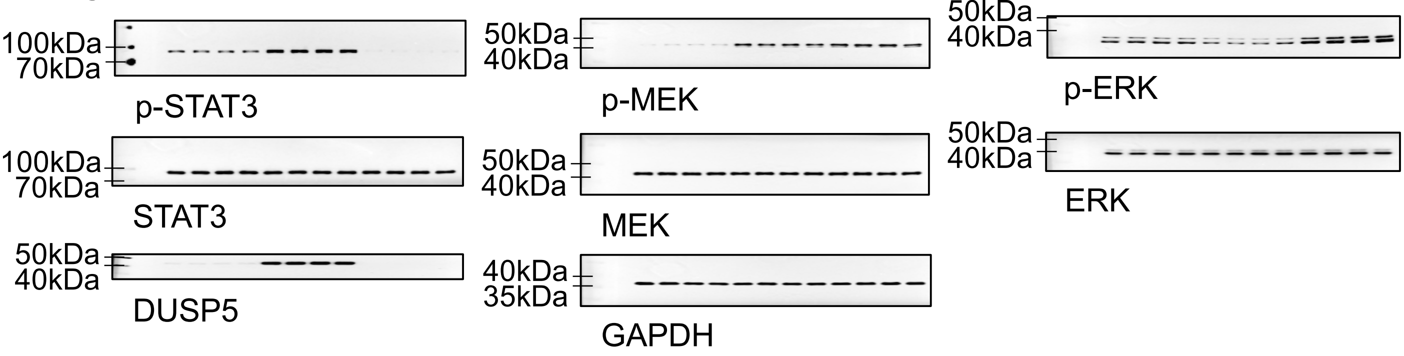

Figure 2b

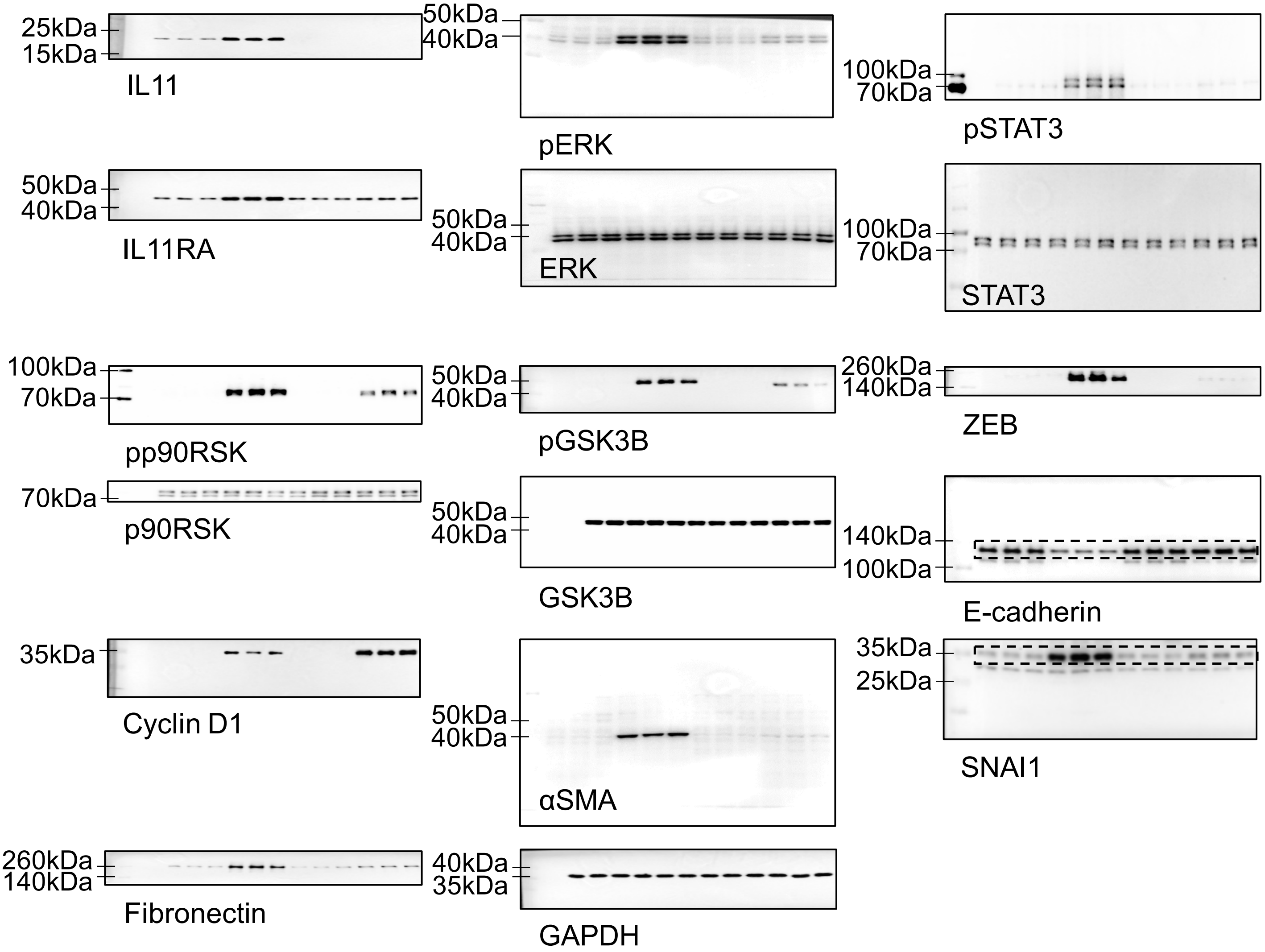

Figure 3l

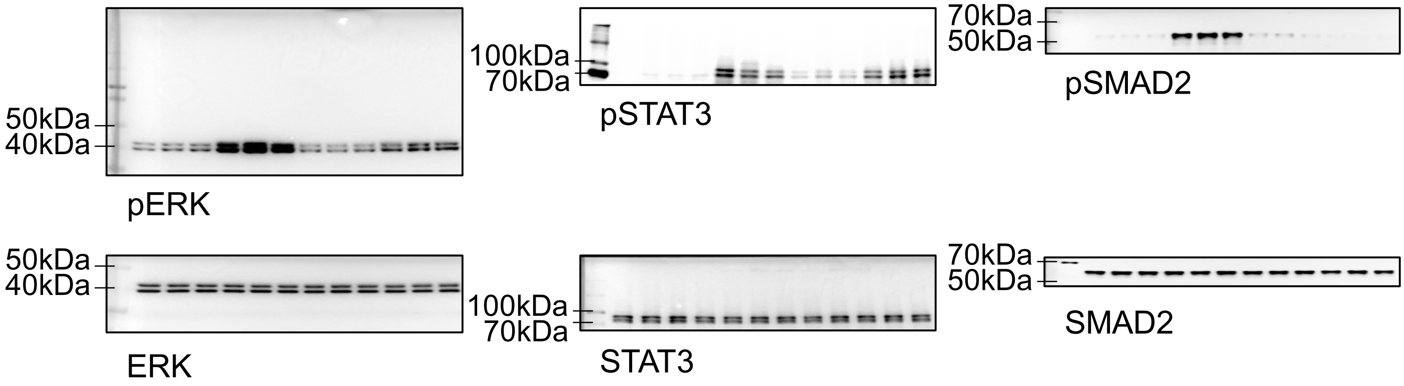

Figure 4d

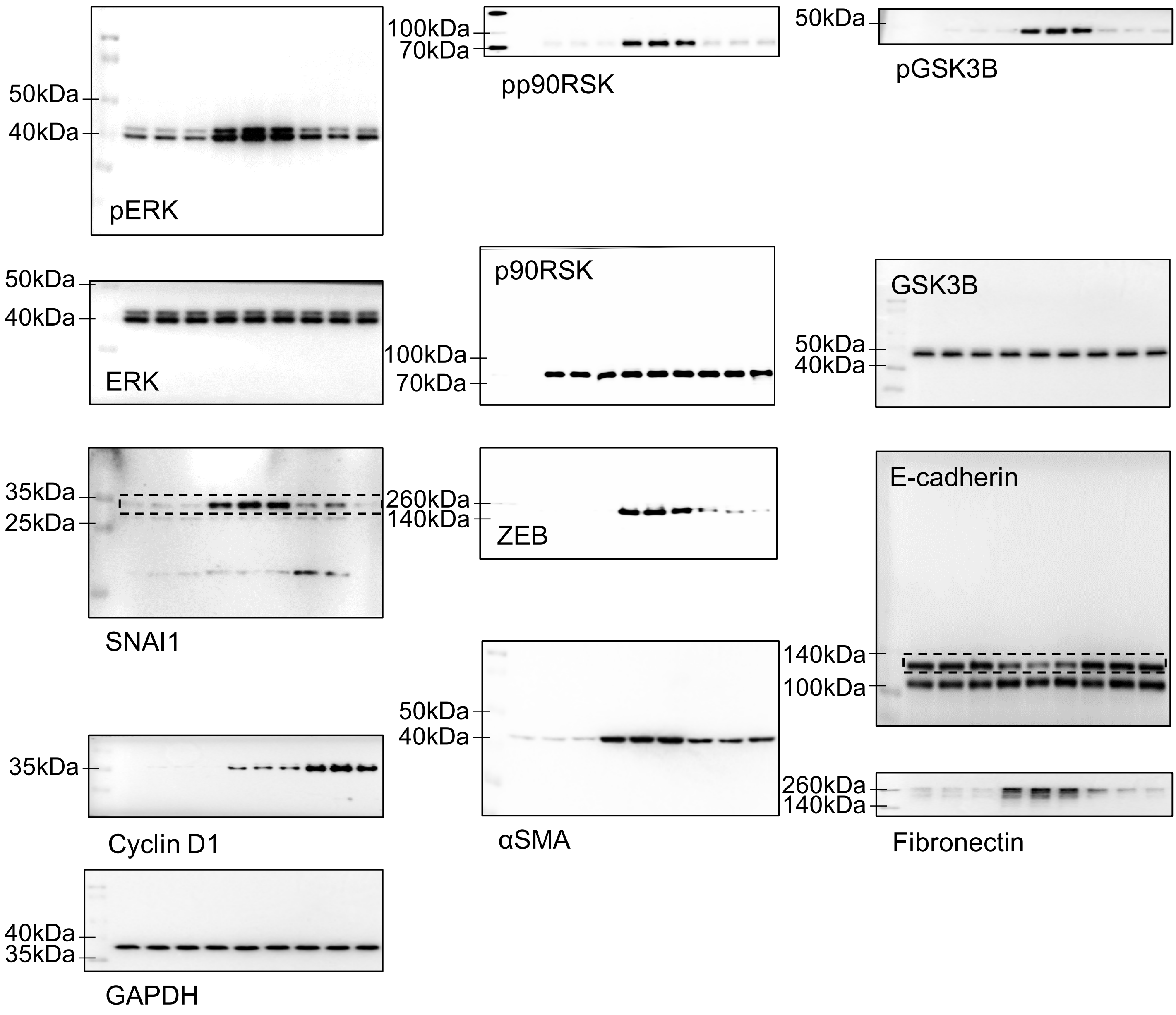

50kDa
40kDa
pERK
100kDa
70kDa
pp90RSK
50kDa
pGSK3B
50kDa
40kDa
ERK
p90RSK
100kDa
70kDa
GSK3B
50kDa
40kDa
35kDa
25kDa
SNAI1
260kDa
140kDa
ZEB
E-cadherin
140kDa
100kDa
50kDa
40kDa
35kDa
Cyclin D1
αSMA
260kDa
140kDa
Fibronectin
40kDa
35kDa
GAPDH

Figure 5b

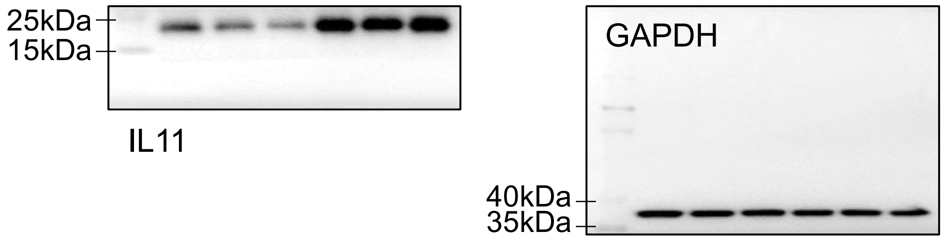

Figure 5d

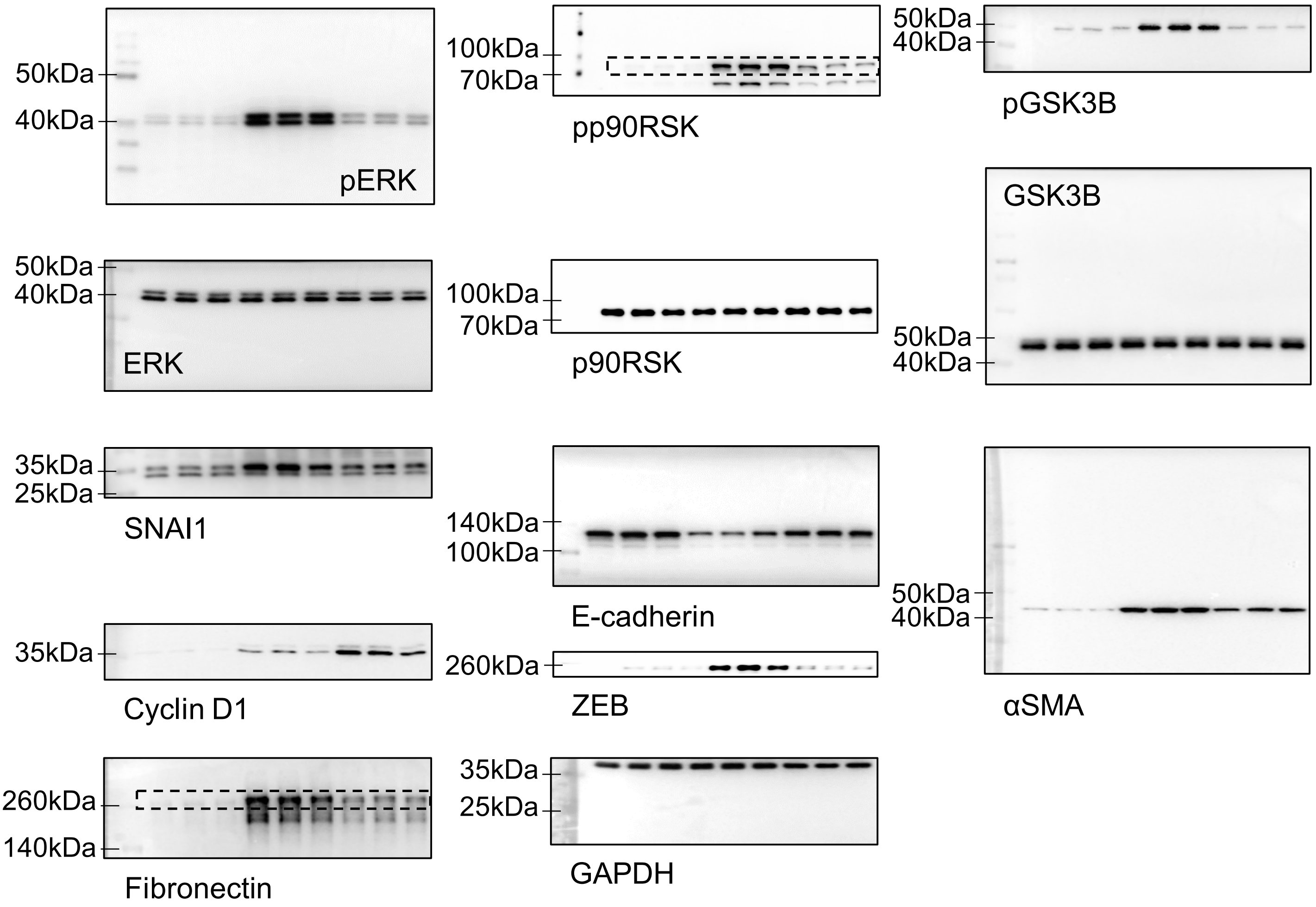

Figure 6b

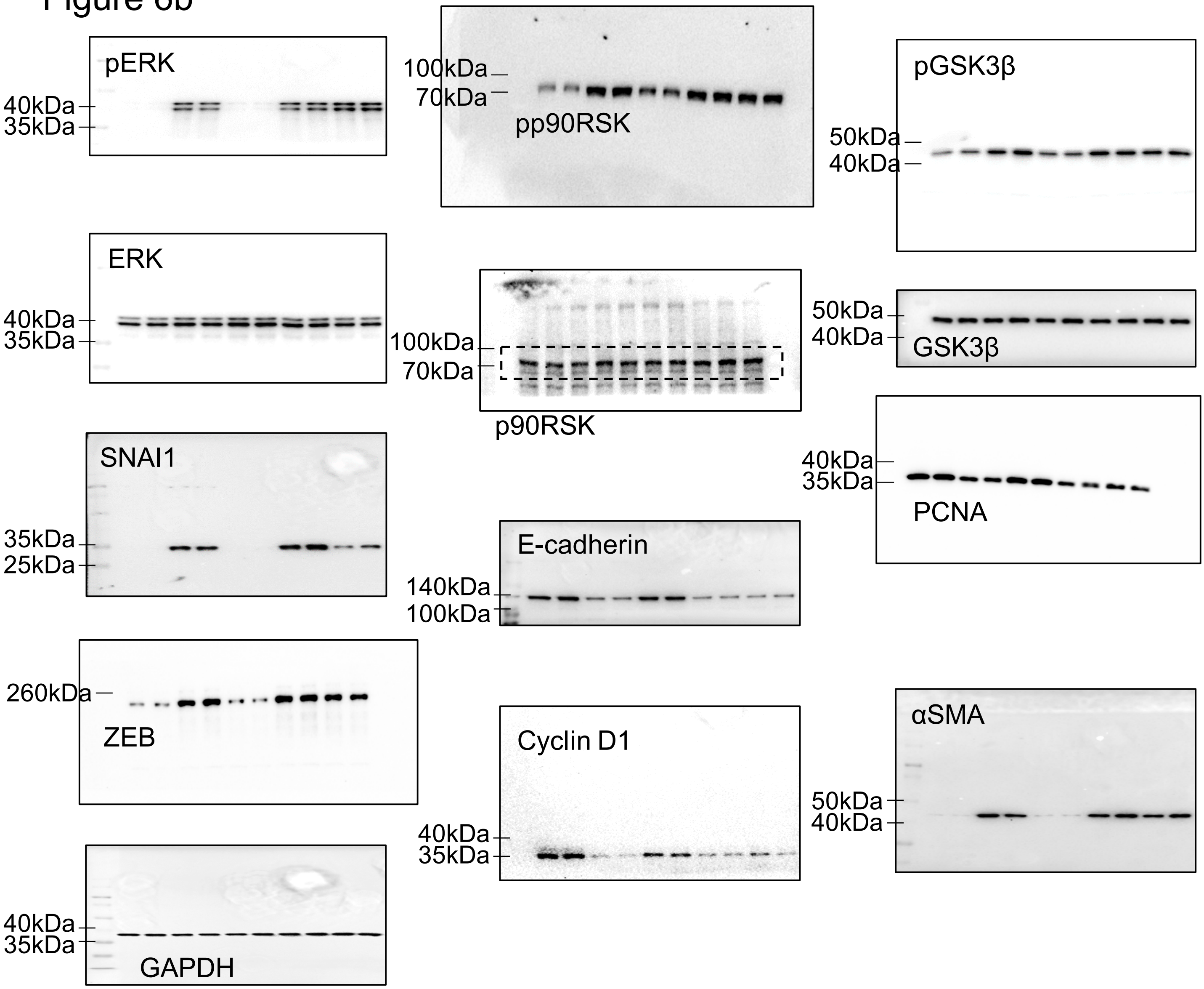

pERK
40kDa
35kDa
ERK
40kDa
35kDa
SNAI1
35kDa
25kDa
ZEB
260kDa
GAPDH
40kDa
35kDa
100kDa
70kDa
pp90RSK
100kDa
70kDa
p90RSK
E-cadherin
140kDa
100kDa
Cyclin D1
40kDa
35kDa
pGSK3β
50kDa
40kDa
50kDa
40kDa
GSK3β
40kDa
35kDa
PCNA
αSMA
50kDa
40kDa

Figure 6d

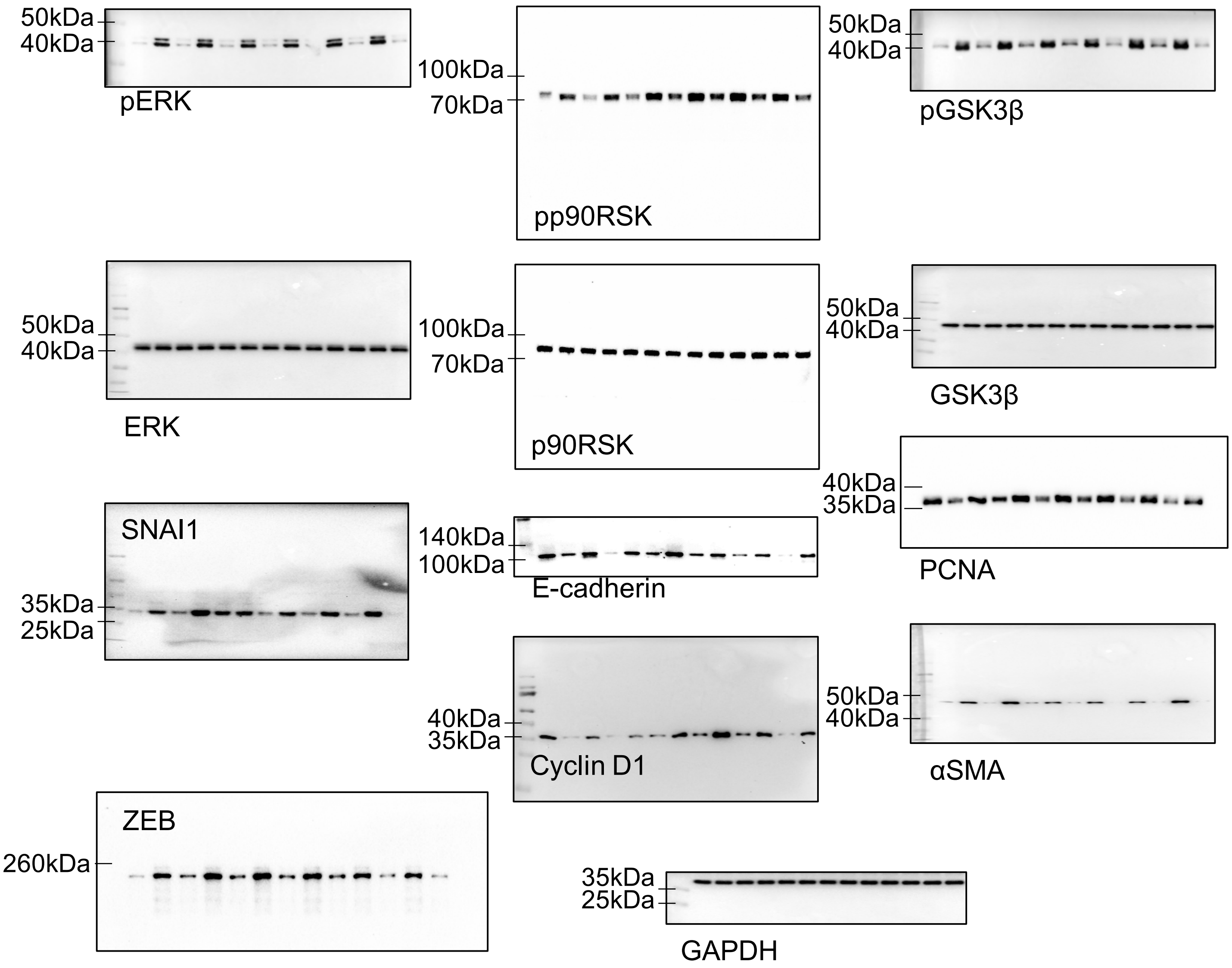

50kDa
40kDa
pERK
100kDa
70kDa
pp90RSK
50kDa
40kDa
pGSK3β
50kDa
40kDa
ERK
100kDa
70kDa
p90RSK
50kDa
40kDa
GSK3β
40kDa
35kDa
PCNA
SNAI1
35kDa
25kDa
140kDa
100kDa
E-cadherin
40kDa
35kDa
Cyclin D1
50kDa
40kDa
αSMA
ZEB
260kDa
35kDa
25kDa
GAPDH

Figure 7b

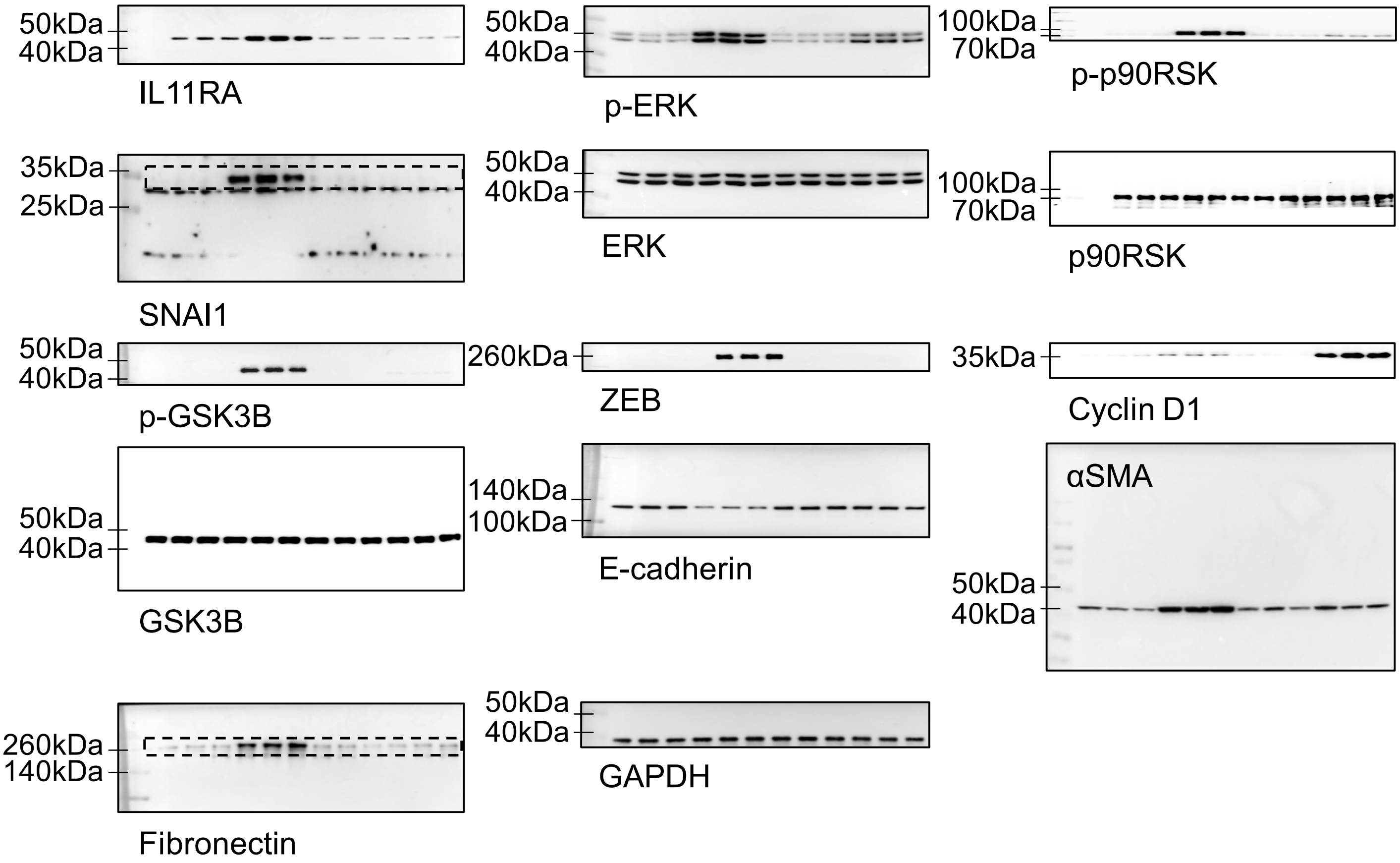
50kDa
40kDa
IL11RA
50kDa
40kDa
p-ERK
100kDa
70kDa
p-p90RSK
35kDa
25kDa
SNAI1
50kDa
40kDa
ERK
100kDa
70kDa
p90RSK
50kDa
40kDa
p-GSK3B
260kDa
ZEB
35kDa
Cyclin D1
50kDa
40kDa
GSK3B
140kDa
100kDa
E-cadherin
αSMA
50kDa
40kDa
260kDa
140kDa
Fibronectin
50kDa
40kDa
GAPDH

Figure 8a

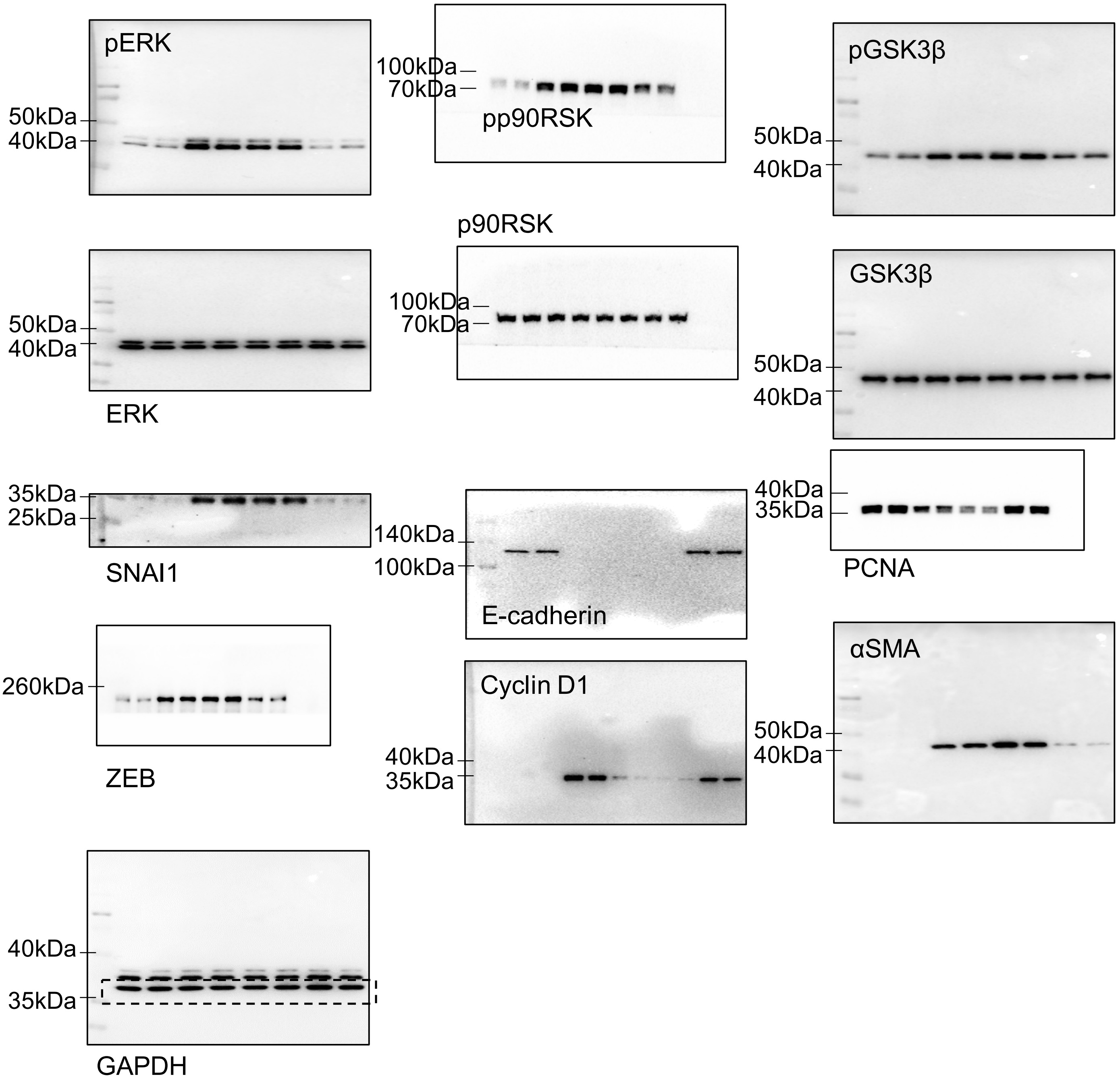
pERK
50kDa
40kDa
pp90RSK
100kDa
70kDa
pGSK3β
50kDa
40kDa
ERK
50kDa
40kDa
p90RSK
100kDa
70kDa
GSK3β
50kDa
40kDa
SNAI1
35kDa
25kDa
E-cadherin
140kDa
100kDa
PCNA
40kDa
35kDa
ZEB
260kDa
Cyclin D1
40kDa
35kDa
αSMA
50kDa
40kDa
GAPDH
40kDa
35kDa

Figure 9f

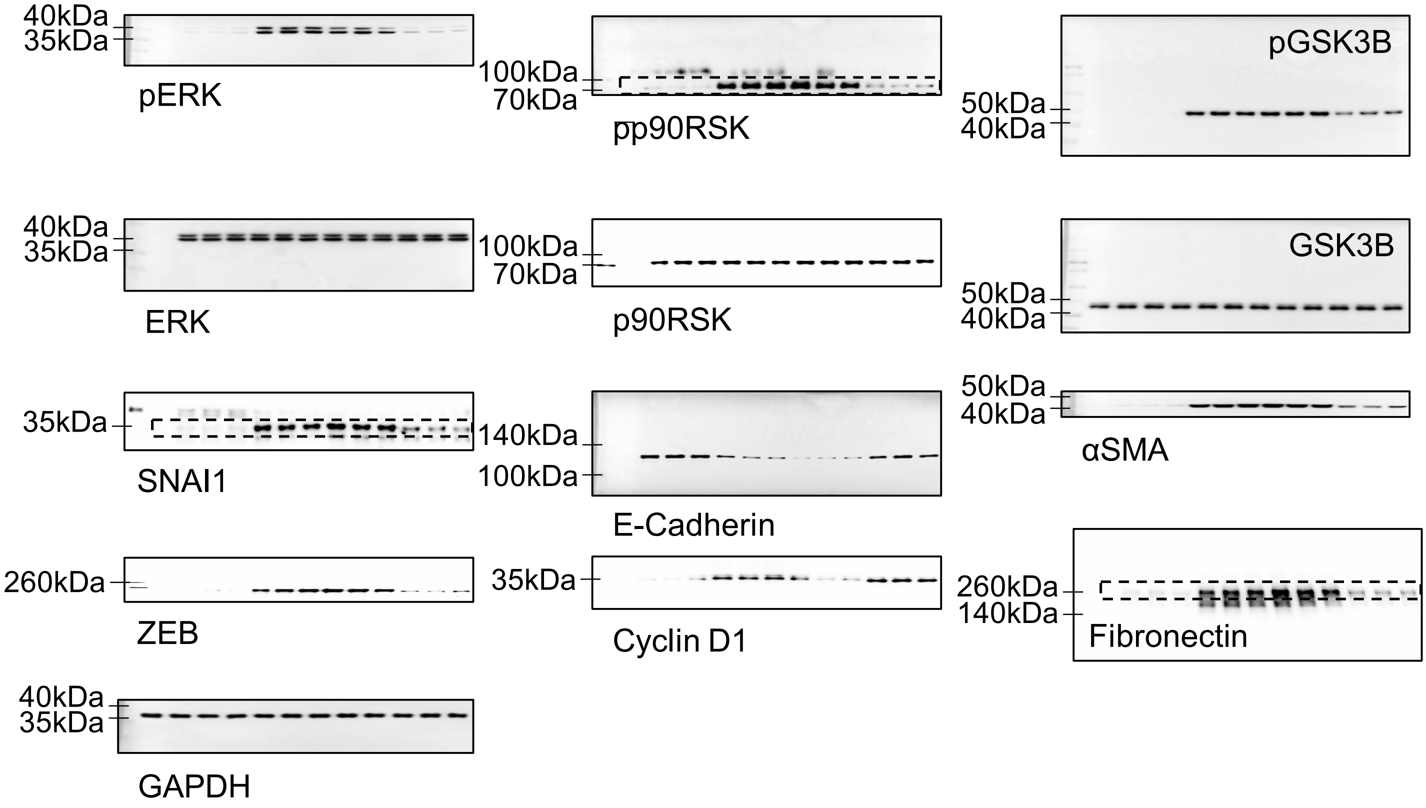

40kDa
35kDa
pERK
100kDa
70kDa
pp90RSK
pGSK3B
50kDa
40kDa
40kDa
35kDa
ERK
100kDa
70kDa
p90RSK
GSK3B
50kDa
40kDa
35kDa
SNAI1
140kDa
100kDa
E-Cadherin
50kDa
40kDa
αSMA
260kDa
ZEB
35kDa
Cyclin D1
260kDa
140kDa
Fibronectin
40kDa
35kDa
GAPDH
