# Supplementary figures and images for: Targeting endogenous kidney regeneration using anti-IL11 therapy in acute and chronic models of kidney disease
Source: Nat Commun. 2022 Dec 5;13:7497. doi: 10.1038/s41467-022-35306-1 (PMC9723120; doi:10.1038/s41467-022-35306-1)

1D

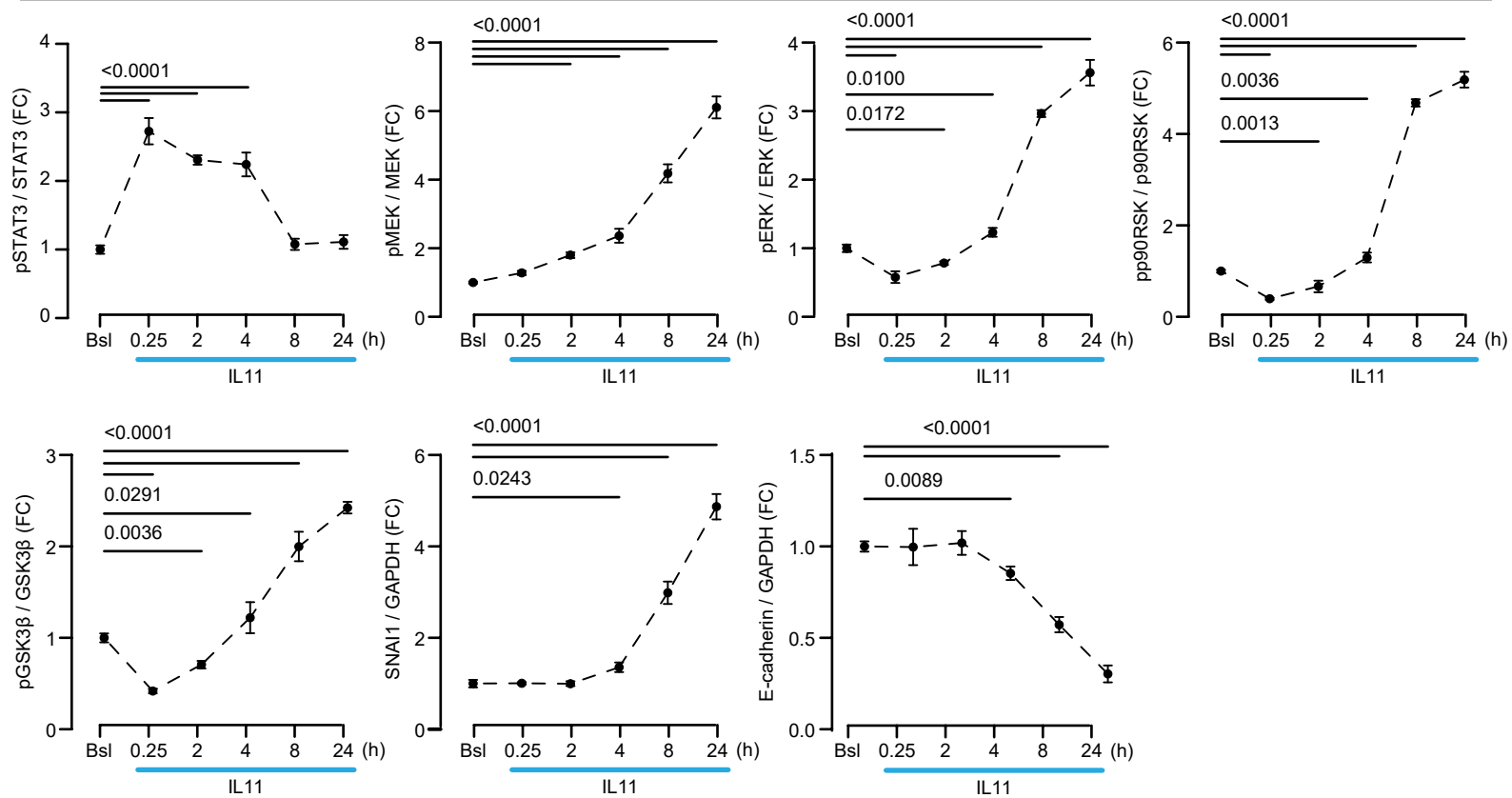

1H

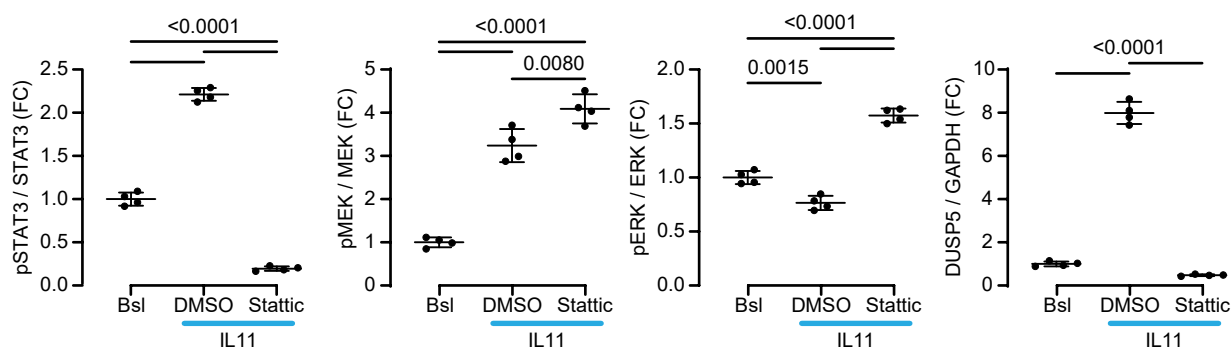

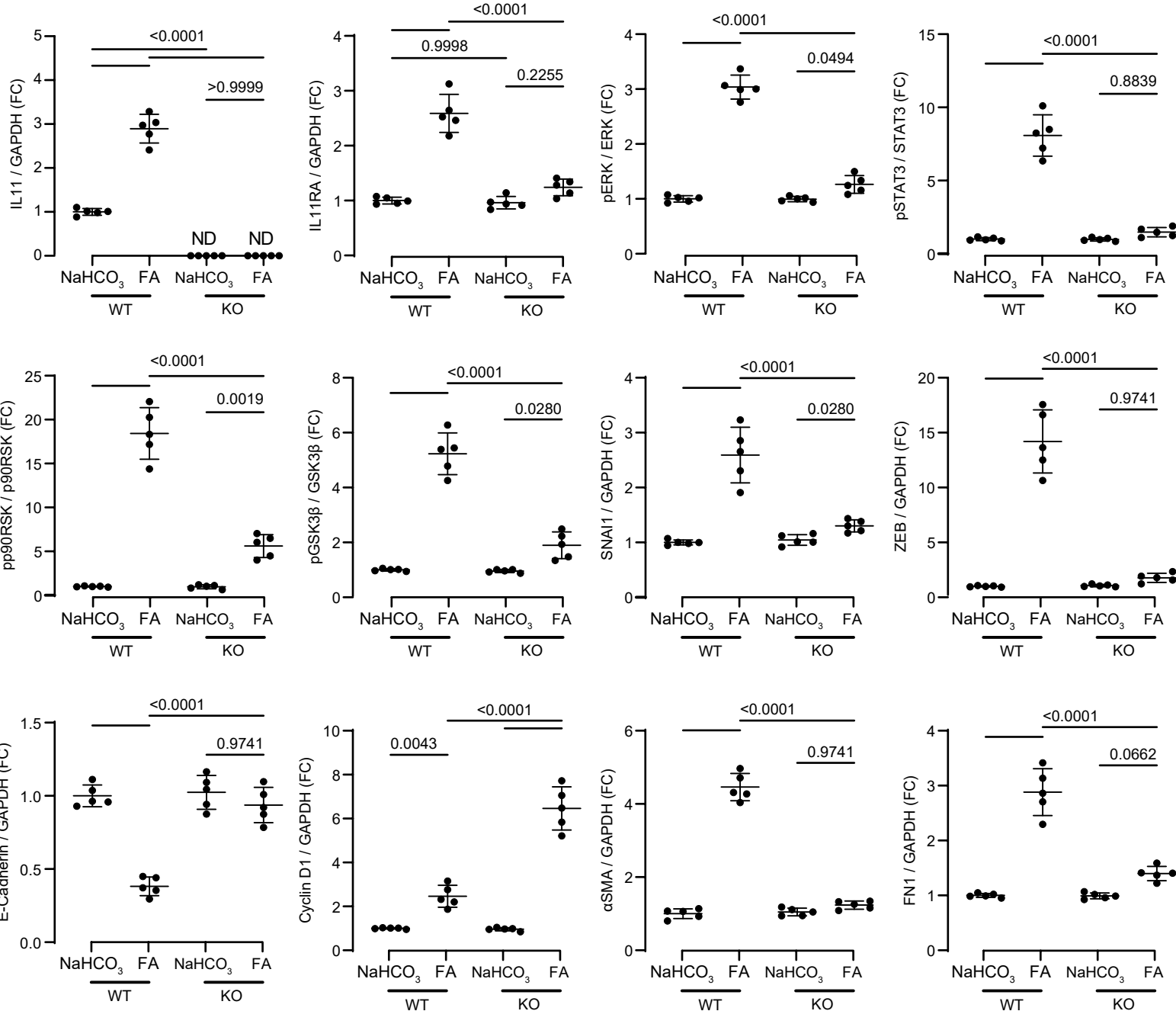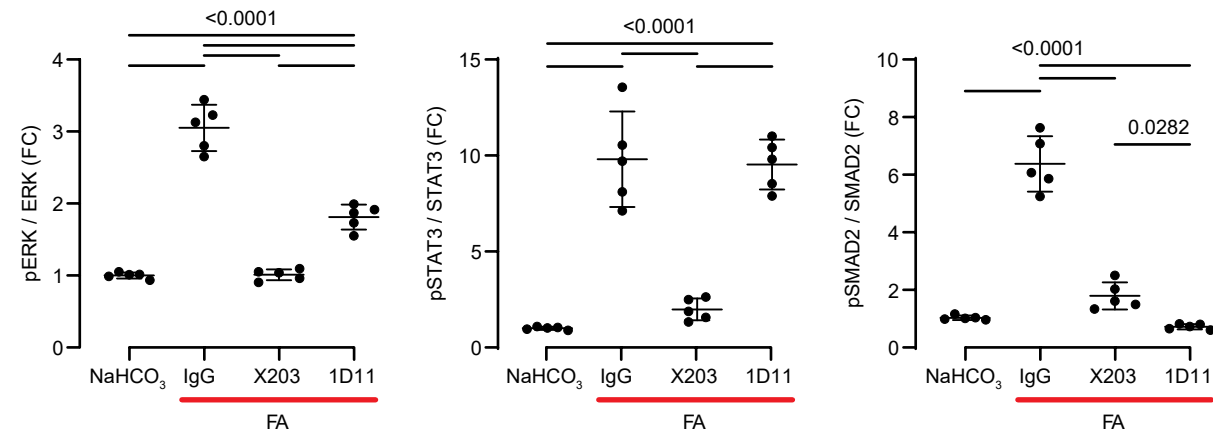

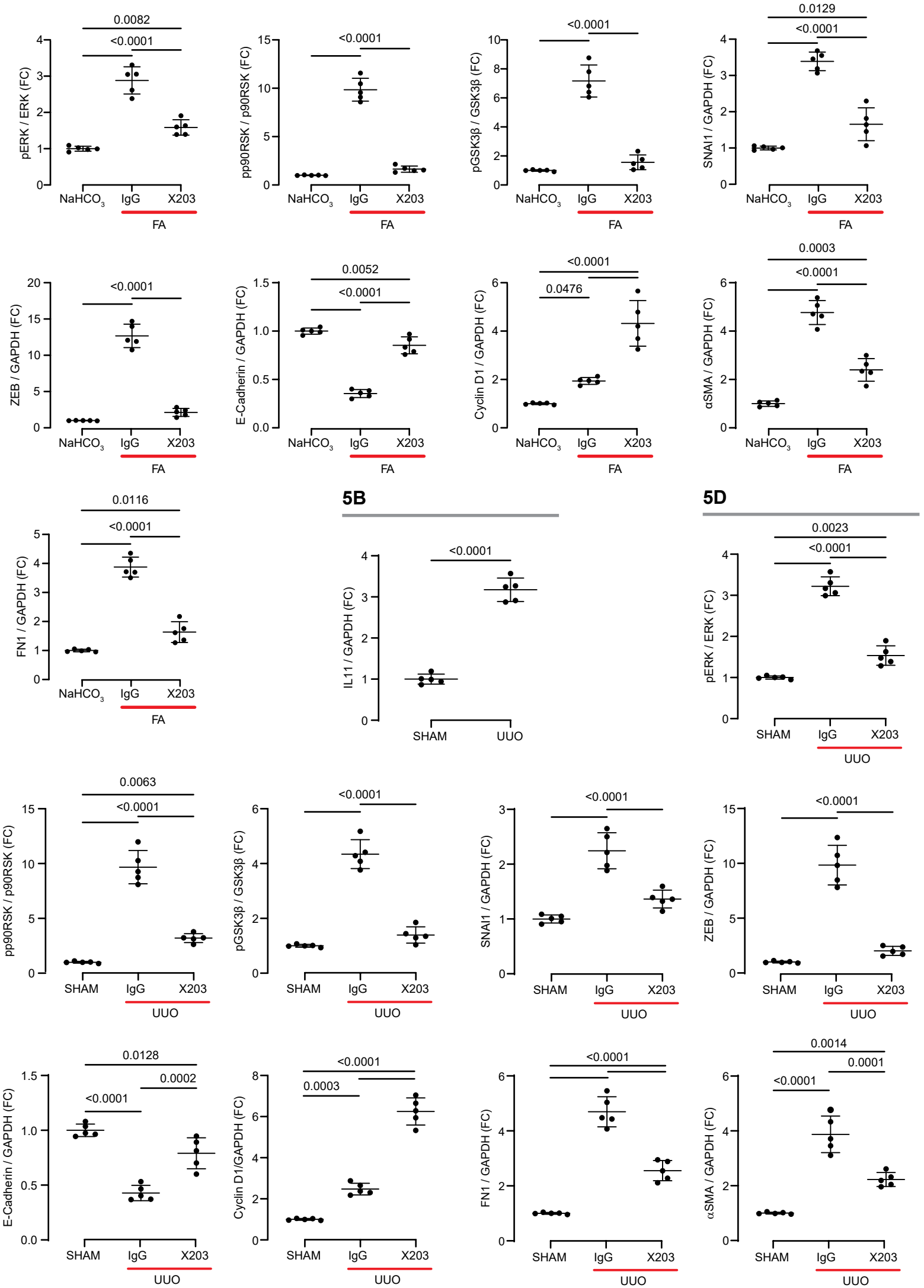

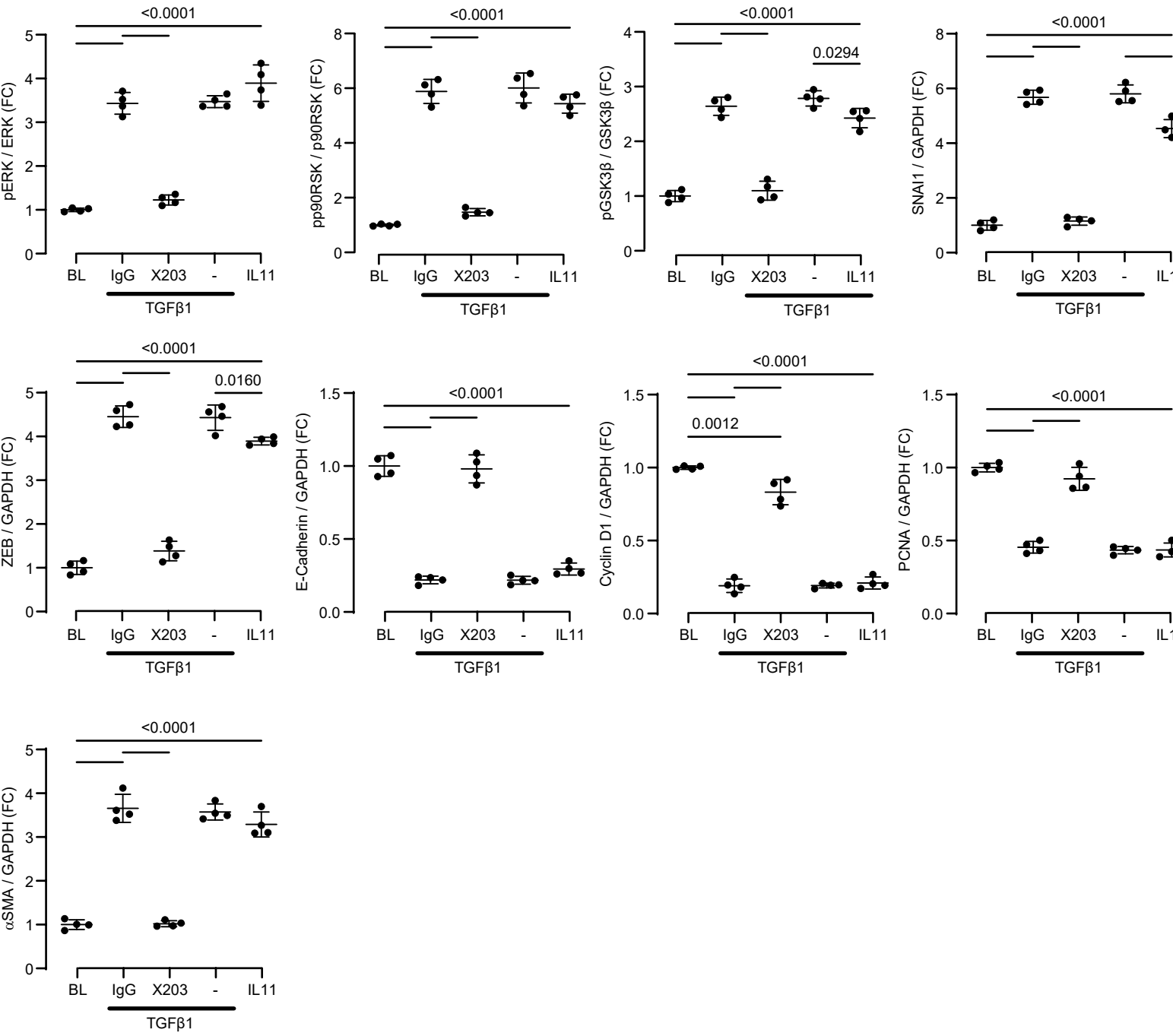

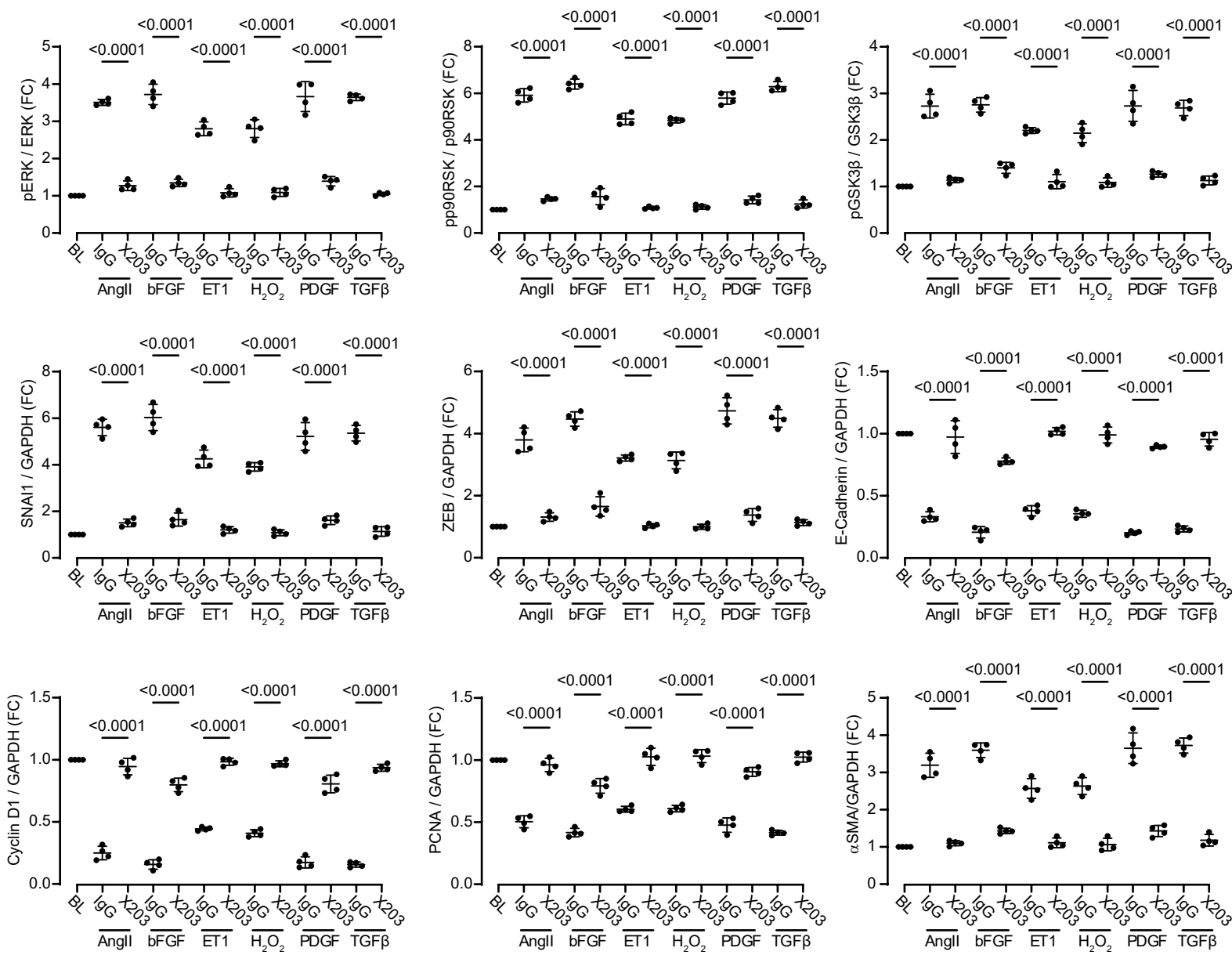

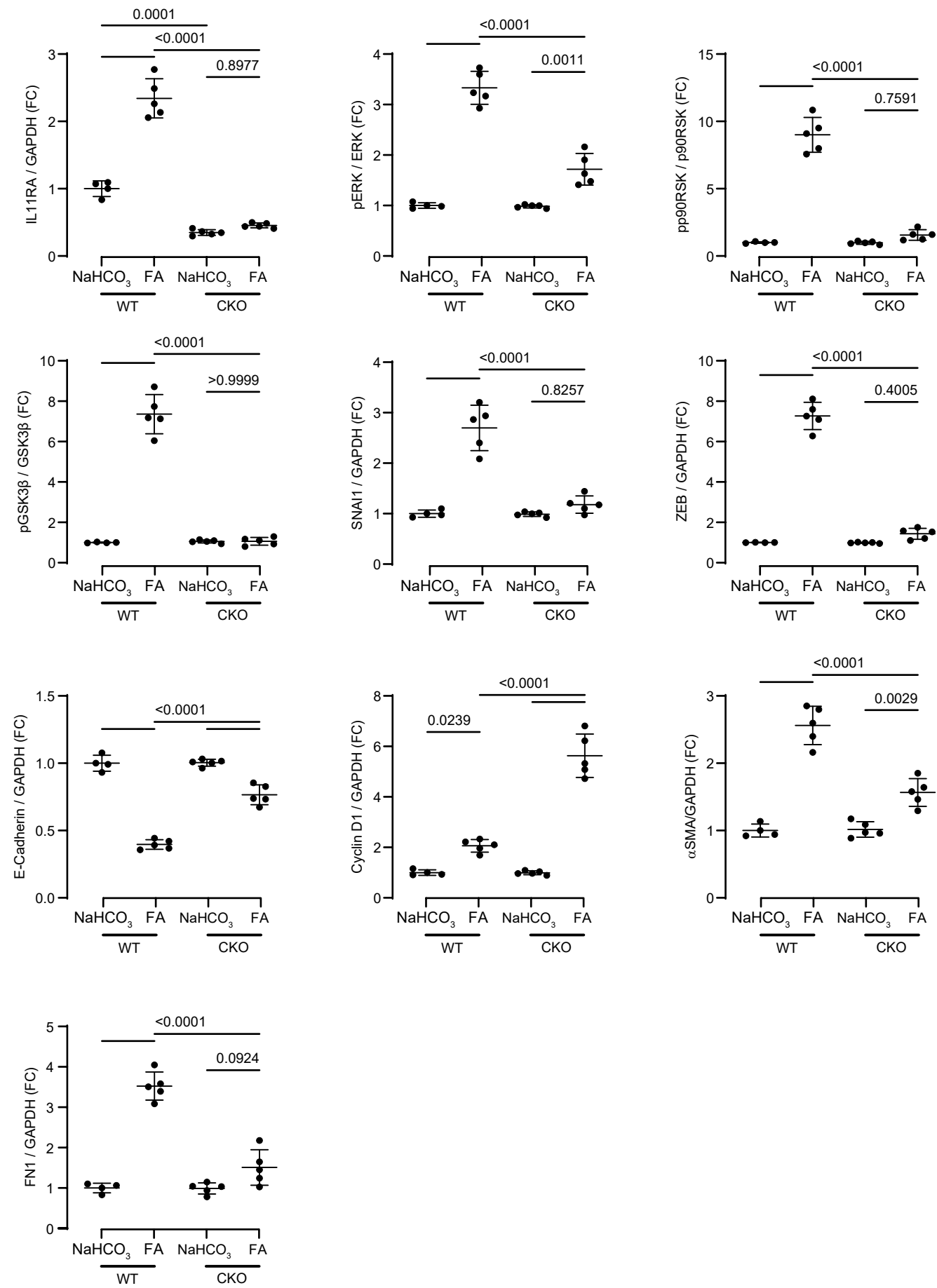

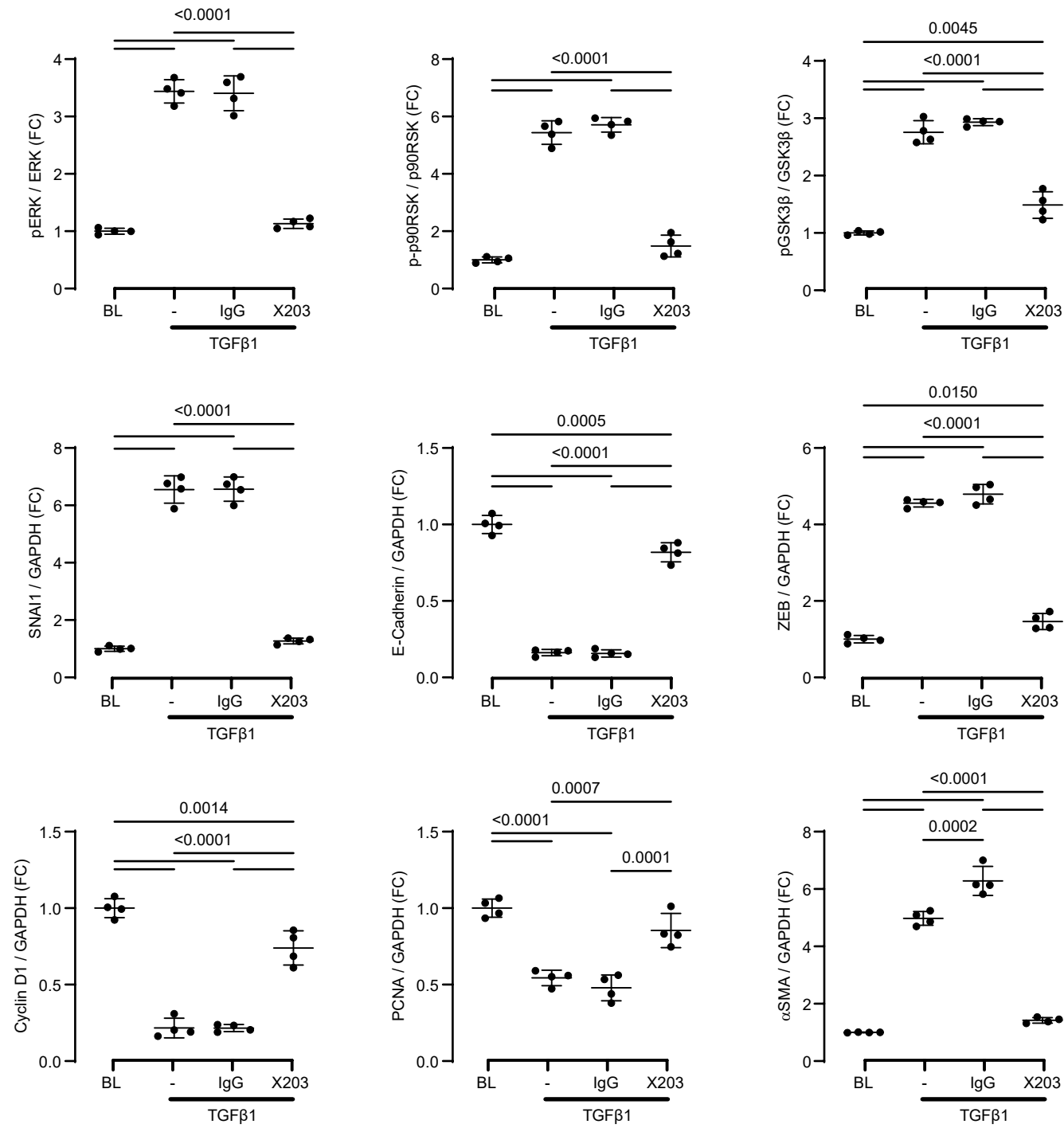

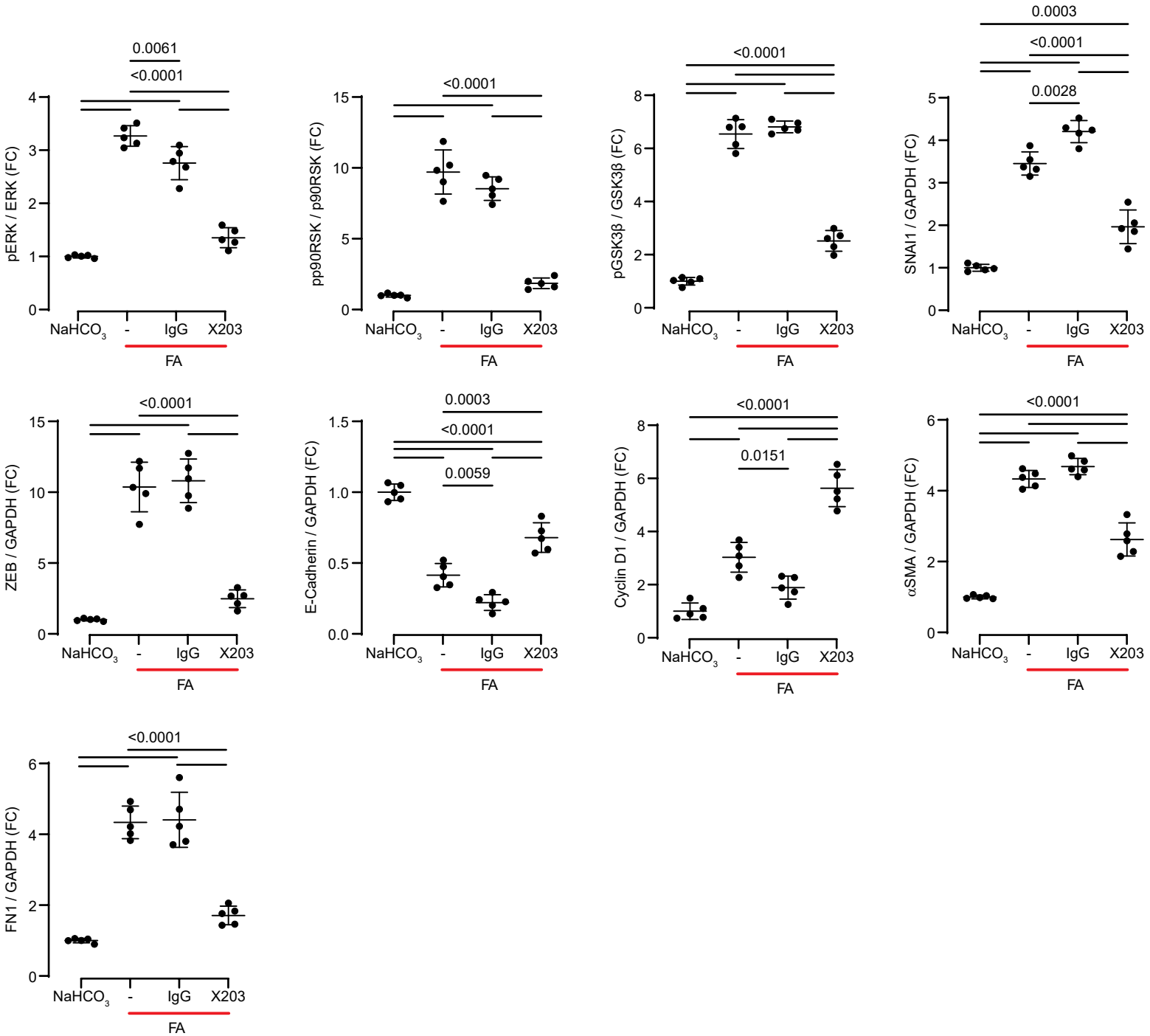

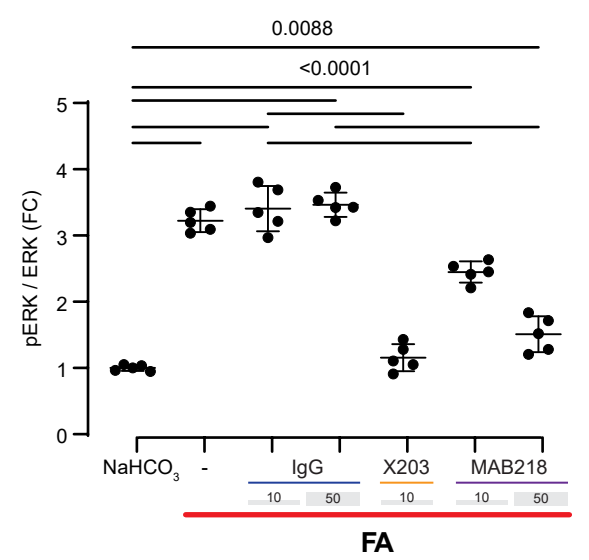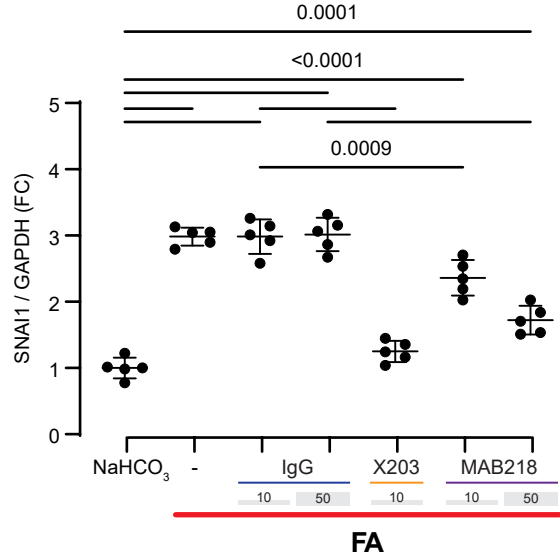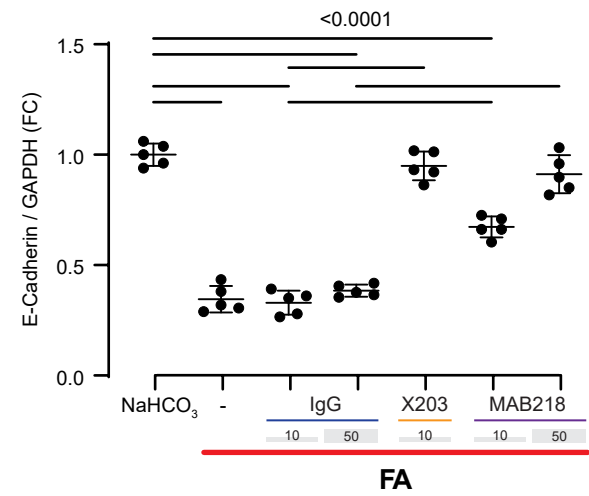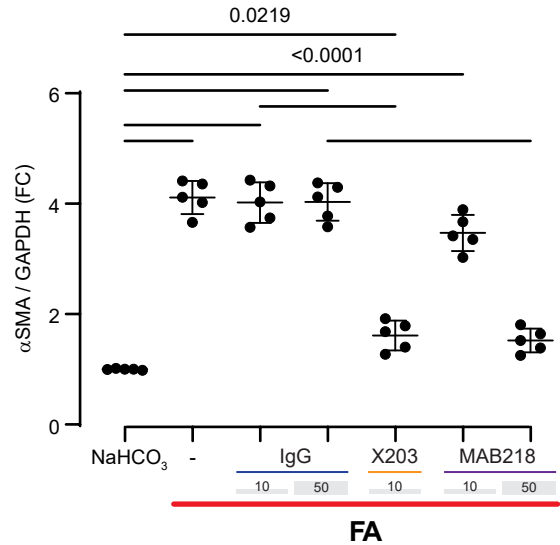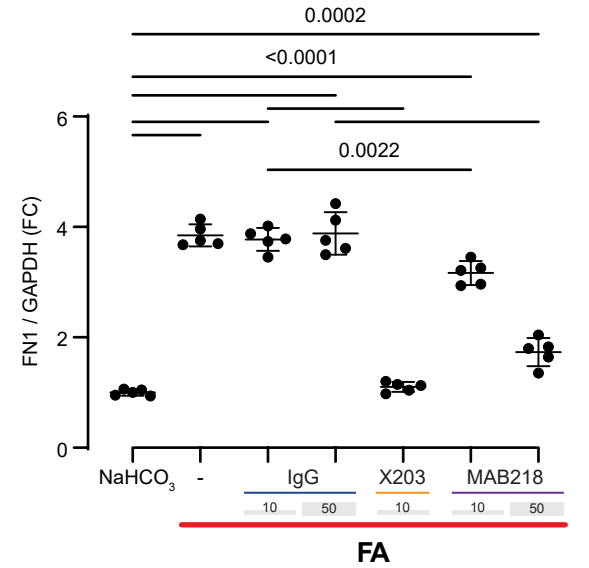

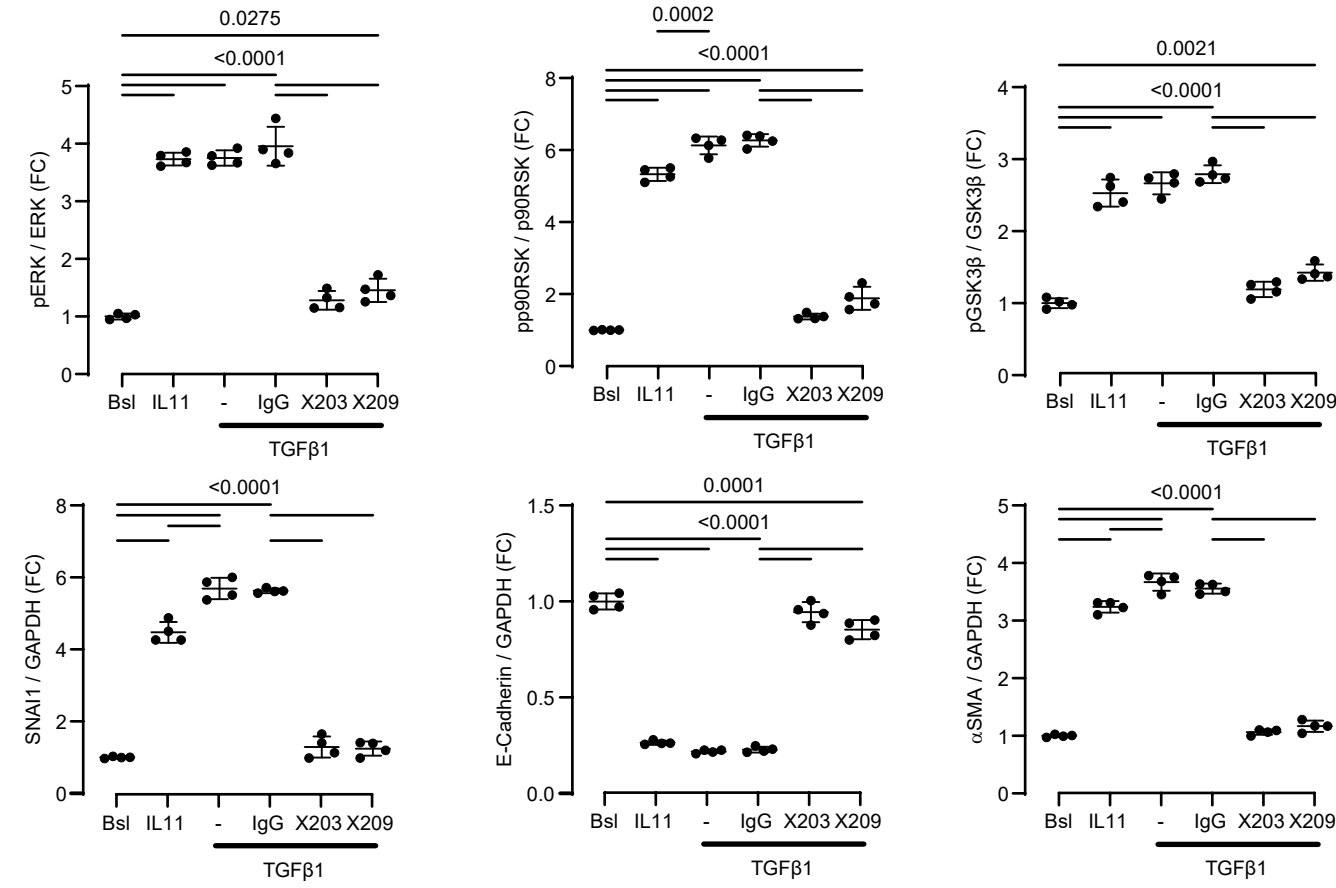

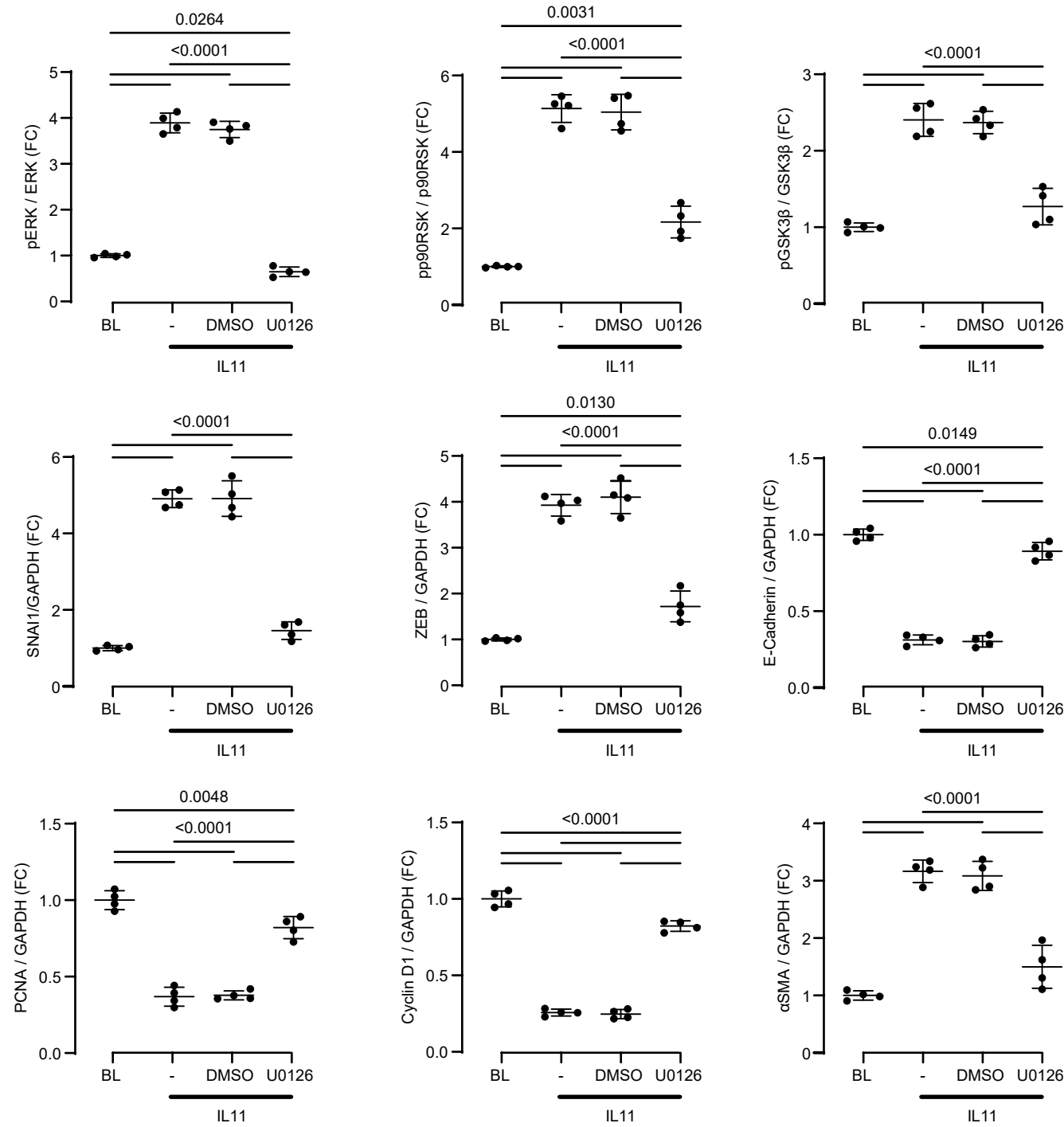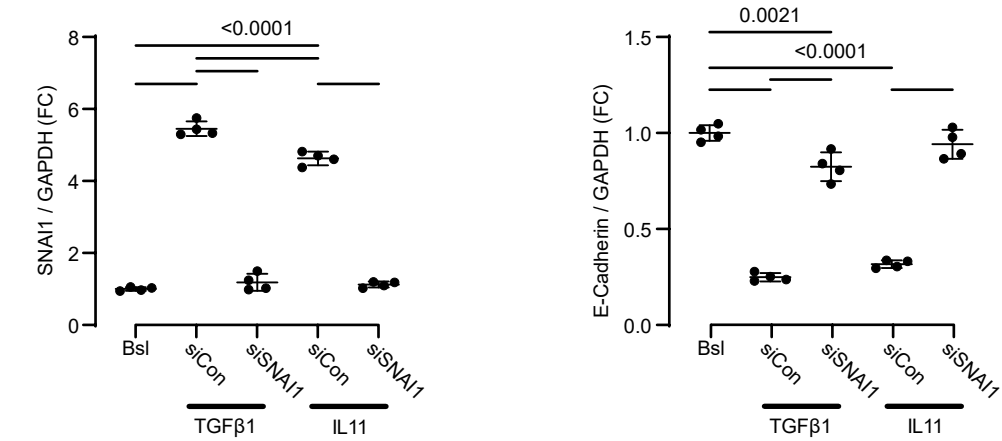

Supplement: Supplementary file 7 — source data file [file 41467_2022_35306_MOESM7_ESM.zip › Source Data file 2.pdf]
